# Metamaterials with amplitude gaps for elastic solitons

**DOI:** 10.1038/s41467-018-05908-9

**Published:** 2018-08-24

**Authors:** Bolei Deng, Pai Wang, Qi He, Vincent Tournat, Katia Bertoldi

**Affiliations:** 1 Harvard John A. Paulson School of Engineering and Applied Sciences, Harvard University, Cambridge, MA 02138 USA; 2 School of Aerospace Engineering, Tsinghua University, 100084 Beijing, China; 3 LAUM, CNRS, Le Mans Université, Av. O. Messiaen, 72085 Le Mans, France; 4 Kavli Institute, Harvard University, Cambridge, MA 02138 USA

## Abstract

We combine experimental, numerical, and analytical tools to design highly nonlinear mechanical metamaterials that exhibit a new phenomenon: gaps in amplitude for elastic vector solitons (i.e., ranges in amplitude where elastic soliton propagation is forbidden). Such gaps are fundamentally different from the spectral gaps in frequency typically observed in linear phononic crystals and acoustic metamaterials and are induced by the lack of strong coupling between the two polarizations of the vector soliton. We show that the amplitude gaps are a robust feature of our system and that their width can be controlled both by varying the structural properties of the units and by breaking the symmetry in the underlying geometry. Moreover, we demonstrate that amplitude gaps provide new opportunities to manipulate highly nonlinear elastic pulses, as demonstrated by the designed soliton splitters and diodes.

## Abstract (web summary)

Here, the authors experimentally observed, numerically simulate, and mathematically analyze the existence of amplitude gaps for elastic vector solitons in highly deformable mechanical metamaterials consisting of rigid units and elastic hinges.

## Introduction

Following John Scott Russell’s observation of nonlinear water wave packets propagating with stable shape and constant velocity in the Union Canal in Scotland^1^, the unique properties of solitons have been studied and exploited in many areas of science and engineering^2–4^. Focusing on mechanical systems, granular crystals have been found to provide an effective platform for the propagation of highly nonlinear solitary waves^4–7^ and have enabled the design of impact mitigation layers^8^, lenses^9^, switches^10^, and non-destructive detection techniques^11^. However, the solitons observed in granular media are of scalar nature and lack the multiple polarizations typical of elastic waves propagating in solid materials.

Polarization is an important property of vector waves like electromagnetic and elastic waves. The ability to control the polarization of light has enabled a broad range of applications, including optical communications, spectroscopy, and microscopy^3,12,13^. Moreover, a broad range of new functionality has been observed in elastic systems with architecture designed to manipulate both the longitudinal and shear polarizations of linear elastic waves^14,15^. While the field initially focused on linear elastic vibrations, it has been recently shown that highly deformable mechanical metamaterials can support elastic vector solitons with two polarizations—one translational and one rotational^16^, but the potential of such solitary waves in applications is unknown and remains to be explored.

Here, we combine experimental, numerical, and analytical tools to demonstrate that elastic vector solitons provide unique opportunities to manipulate the propagation of large amplitude vibrations. Specifically, we show that in mechanical metamaterials based on rotating rigid units, both the amplitude of the propagating waves and the symmetry of the underlying building blocks can be used to significantly alter the coupling between the two polarizational components of the vector solitons. We find that such control of the coupling strength results in the emergence of a new phenomenon: the formation of amplitude gaps for solitons. Notably, this new effect can be exploited to realize devices capable of controlling and manipulating the propagation of large amplitude vibrations in unprecedented ways, as demonstrated by the design of soliton splitters and diodes.

## Results

### Metamaterial design and characterization

**Fig. 1:**
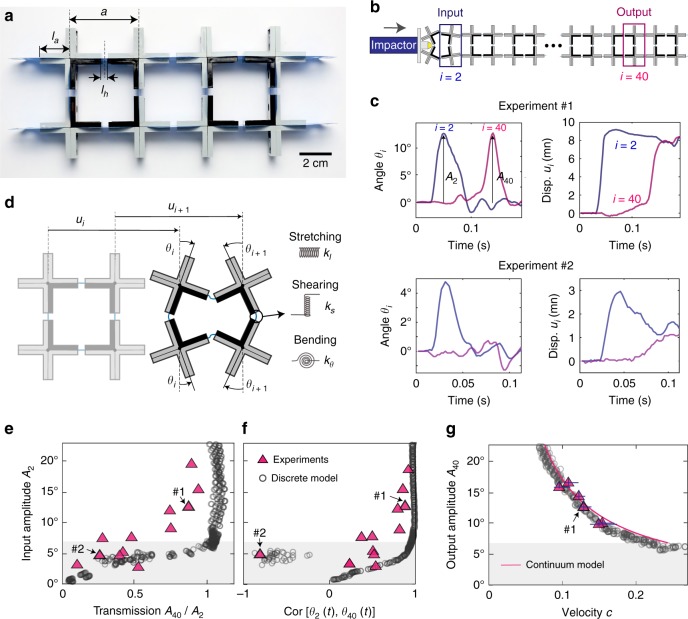
Propagation of elastic vector solitons in a chain with all horizontal hinges aligned. **a** Few units of our sample (Scale bar: 2 cm). **b** Schematics of our testing setup. **c** Evolution of the rotation and longitudinal displacement of the second and fortieth units as a function of time during two different experiments. **d** Schematic of the system. **e** Measured transmission, *A*_40_/*A*_2_, as a function of the amplitude of the input signal, *A*_2_. **f** Measured cross-correlation of *θ*_2_(*t*) and *θ*_40_(*t*) as a function of the amplitude of the input signal, *A*_2_. **g** Evolution of the pulse velocity *c* as a function of its amplitude. The gray region in **e**–**g** highlights the amplitude gap as predicted by the continuum model. The error bars in **g** show the 95% confident interval of the measured velocities and amplitudes of solitons in experiments. The corresponding error bars for simulation results are too small to show

Our system consists of a long chain of 2 × 50 rigid crosses made of LEGO bricks^17^ with arm length *l*_*a*_ = 19 mm connected by thin and flexible hinges made of polyester plastic sheets (Artus Corporation, NJ) with length *l*_*h*_ = 4 mm and thickness *t*_*h*_ = 0.127 mm, resulting in a spatial periodicity *a* = 2*l*_*a*_ + *l*_*h*_ = 42 mm (Fig. 1a—see Supplementary Note 1 and Supplementary Movie 1 for details on fabrication). To investigate the propagation of elastic pulses in the system, we place the chain (supported by pins to minimize frictions) on a smooth horizontal surface and use an impactor excited by a pendulum to hit the mid-point at its left end (see Fig. 1b-top and Supplementary Movie 2). We apply different input signals to the chain by varying both the initial height of the striking pendulum and the distance traveled by the impactor and find that all of them initiate simultaneous rotation and displacement of the rigid units, with each pair of crosses in a column sharing the same displacement and rotating by the same amount, but in opposite directions (i.e., if the top unit rotates by a certain amount in clockwise direction, then the bottom one rotates by the same amount in counter-clockwise direction, and vice versa). To monitor the displacement, *u*_*i*_, and rotation, *θ*_*i*_, of the *i*-th pair of crosses along the chain as the pulse propagates, we use a high speed camera (SONY RX100V) and track markers via digital image processing (see Supplementary Note 2).

In Fig. 1c, we report the evolution of the rotation and longitudinal displacement of the second and fortieth pairs of crosses as a function of time during two different experiments. We find that when the amplitude of the input signal is large (*A*_2_ = max(*θ*_2_(*t*)) = 13° in experiment #1) the pulse that propagates through the system conserves its amplitude and shape in both degrees of freedom. Differently, for inputs with small amplitude (*A*_2_ = 5° in experiment #2), the output signal is severely distorted compared to the input one (see Supplementary Movie 3). While in Fig. 1c we focus on two representative experiments, all our experimental results are summarized as triangular markers in Fig. 1e, f, where we present the measured transmission, *A*_40_/*A*_2_ (with *A*_40_ = max(*θ*_40_(*t*))), and cross-correlation of *θ*_2_(*t*) and *θ*_40_(*t*) as a function of the amplitude of the input signal, *A*_2_. We find that if *A*_2_ ≳ 7° both the transmission and the cross-correlation approach unity, suggesting that for large enough input signals, the system supports the propagation of elastic vector solitons. However, for input amplitudes below ~7° a transition occurs and both the transmission and the cross-correlation significantly and systematically decrease. This indicates that our system might only support the propagation of solitary waves with amplitude above a certain threshold, manifesting a gap in amplitude for 0° ≲ *A*_2_ ≲ 7°.

### Discrete and continuum models

To better understand these experimental results, we establish a discrete model in which the crosses are represented as rigid bodies of mass *m* and rotational inertia *J*. Guided by our experiments, we assume that the system has an horizontal line of symmetry and assign two degrees of freedom (*u*_*i*_ and *θ*_*i*_) to the top unit of the *i*-th pair of crosses (Fig. 1d). As for the flexible hinges, they are modeled using a combination of three linear springs: their stretching is captured by a spring with stiffness *k*_*l*_; their shearing is described by a spring with stiffness *k*_*s*_; their bending is modeled by a torsional spring with stiffness *k*_*θ*_. Under these assumptions, the dimensionless equations of motion for the *i*-th top unit are given by (see Supplementary Note 3)

1 $$\begin{array}{*{20}{l}} {\frac{{\partial ^2U_i}}{{\partial T^2}}} \hfill & = \hfill & {U_{i + 1} - 2U_i + U_{i - 1} - {\mathrm{cos}}{\kern 1pt} \theta _{i + 1} + {\mathrm{cos}}{\kern 1pt} \theta _{i - 1},} \hfill \\ {\frac{1}{{\alpha ^2}}\frac{{\partial ^2\theta _i}}{{\partial T^2}}} \hfill & = \hfill & - K_\theta \left( {\theta _{i + 1} + 4\theta _i + \theta _{i - 1}} \right) \hfill \\ {} \hfill & {} \hfill & + \hskip1pt K_s{\mathrm{cos}}{\kern 1pt} \theta _i\left[ {{\mathrm{sin}}{\kern 1pt} \theta _{i + 1} + {\mathrm{sin}}{\kern 1pt} \theta _{i - 1} - 2{\kern 1pt} {\mathrm{sin}}{\kern 1pt} \theta _i} \right] \hfill \\ {} \hfill & {} \hfill & - \hskip1pt {\mathrm{sin}}{\kern 1pt} \theta _i\left[ 2\left( {U_{i + 1} - U_{i - 1}} \right) + 4 - {\mathrm{cos}}{\kern 1pt} \theta _{i + 1}\right. \hfill \\ {} \hfill & {} \hfill & \left. - \hskip1pt 2 {\kern 1pt} {\mathrm{cos}}{\kern 1pt} \theta _i - {\mathrm{cos}}{\kern 1pt} \theta _{i - 1} \right], \hfill \end{array}$$

where *U*_*i*_ = *u*_*i*_/*a*, *T* = $t\sqrt {k_l{\mathrm{/}}m}$, *K*_*θ*_ = $4k_\theta {\mathrm{/}}\left( {k_la^2} \right)$, *K*_*s*_ = *k*_*s*_/*k*_*l*_ and *α* = $a\sqrt {m{\mathrm{/}}(4J)}$ are all non-dimensional parameters, which in our system are measured as *K*_*s*_ = 0.02, *K*_*θ*_ = 1.5 × 10^−4^, and *α* = 1.8. Note that, as shown in Fig. 1d, to facilitate the analysis in our model we define the positive direction of rotation alternatively for neighboring crosses (i.e., if for the *i*-th top unit a clockwise rotation is positive, then for the (*i* − 1)-th and (*i* + 1)-th ones counterclockwise rotation is positive).

We start by numerically solving Eq. (1) using the Runge–Kutta method (the code implemented in MATLAB is available online). In our numerical analysis we consider a chain comprising 150 pairs of crosses, apply a longitudinal displacement with the form *U*_input_ = *b* + *b* tanh[(*T* − *T*_0_)/*w*] to the mid-point at its left end (see Supplementary Note 3), and implement free-boundary conditions at its right end. In Fig. 1e, f we report as gray circular markers the results of 480 analyses in which we systematically change the applied displacement *U*_input_ (with *b* ∈ [0, 0.75], *w* ∈ [50, 100] and *T*_0_ = 400). In good agreement with our experimental data, we find that for *A*_2_ ≲ 6° the signal does not preserve its amplitude and shape as it propagates through the structure. As such, these numerical results also point to the existence of an amplitude gap for solitons.

### Amplitude gaps for solitons

To confirm the existence of such amplitude gap, we further simplify Eq. (1) to obtain an analytical solution. To this end, we assume that the wavelength of the propagating waves is much wider than the cell size and that $\theta \ll 1$, take the continuum limit of Eq. (1) and retain nonlinear terms up to the third order, obtaining (see Supplementary Note 4)

2 $$\begin{array}{l}\frac{{\partial ^2U}}{{\partial T^2}} = \frac{{\partial ^2U}}{{\partial X^2}} + \theta \frac{{\partial \theta }}{{\partial X}},\\ \frac{1}{{\alpha ^2}}\frac{{\partial ^2\theta }}{{\partial T^2}} = \left( {K_s - K_\theta } \right)\frac{{\partial ^2\theta }}{{\partial X^2}} - 4\left[ {\frac{{3K_\theta }}{2} + \frac{{\partial U}}{{\partial X}}} \right]\theta - 2\theta ^3,\end{array}$$

where *X* = *x*/*a* (*x* denoting the initial position along the chain) and *U*(*X*, *T*) and *θ*(*X*, *T*) are two continuous functions of *X* and *T*. It is easy to show that Eq. (2) admits an analytical solution in the form of an elastic vector soliton with two components^18^

3 $$\left\{ \begin{array}{l}\hskip-3.5pc\theta = A{\kern 1pt} {\mathrm{sech}}\left( {\frac{{X - cT}}{W}} \right),\\ U = \frac{{A^2W}}{{2\left( {1 - c^2} \right)}}\left[ {1 - {\mathrm{tanh}}\left( {\frac{{X - cT}}{W}} \right)} \right],\end{array} \right.$$

where *c* is the pulse velocity, and *A* and *W* are the amplitude and width of the solitary wave, which can be expressed in terms of *c* and the structural parameters as (see Supplementary Note 4)

4 $$A = \pm \sqrt {\frac{{6K_\theta \left( {1 - c^2} \right)}}{{c^2}}} ,\,{\mathrm{and}}\,W = \sqrt {\frac{{\alpha ^2\left( {K_s - K_\theta } \right) - c^2}}{{6\alpha ^2K_\theta }}} .$$

At this point it is important to note that, since the width *W* needs to be real-valued

5 $$c^2 < \alpha ^2\left( {K_s - K_\theta } \right),$$

yielding

6 $$\left| A \right| > A_{{\mathrm{upper}}} = \sqrt {\frac{{6K_\theta }}{{\alpha ^2\left( {K_s - K_\theta } \right)}} - 6K_\theta } .$$

Condition in Eq. (6) clearly indicates that our system has an amplitude gap for solitons, since only solitary waves with amplitude greater than *A*_upper_ = 6.55° are physically admissible solutions. Such amplitude gap is reported as shaded area in Fig. 1e, f and is in excellent agreement with our numerical and experimental results. Note that this gap (and the associated amplitude threshold *A*_upper_ below which solitary waves cannot propagate) is fundamentally different from the nonlinear supratransmission effect (limited to weakly nonlinear periodic waves with certain frequency^19^), classical amplitude-dependent dissipation^20^ and so-called “sonic vacuum” found in not precompressed granular chains^21^. The amplitude gaps for solitons reported in our work are robust features of the system, intrinsically determined by its architecture and its ability to support elastic vector solitons.

Looking into the mechanism behind the emergence of this amplitude gap, it is important to note that the propagation of vector solitons requires a strong coupling among different polarizations^22,23^. However, Eq. (2) show that the coefficients of the coupling terms in our structure are proportional to *θ*, so that large enough rotations are needed in order to activate them and enable the propagation of vector solitons (see Supplementary Note 5). Finally, to further verify the validity of our continuum model, in Fig. 1g we compare the relation between *A* and *c* as predicted by our analysis (magenta line) and measured in our experiments (triangular markers) and numerical direct simulations (circular markers) and find good agreement among all three sets of data.

### Enhanced tunability via symmetry breaking

**Fig. 2:**
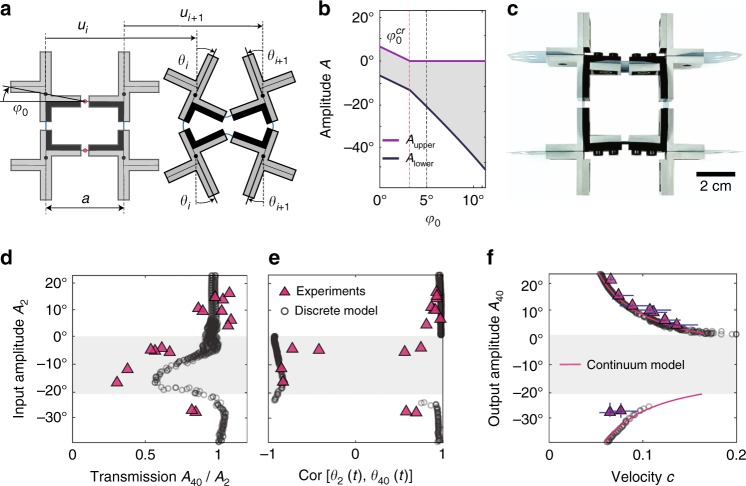
Propagation of elastic vector solitons in a chain with vertically shifted neighboring horizontal hinges. **a** Schematic of the system. Neighboring horizontal hinges are shifted by *a* tan *φ*_0_ in vertical direction. **b** Evolution of the amplitude gap as a function of the angle *φ*_0_. **c** Few units of our sample characterized by *φ*_0_ = 5° (Scale bar: 2 cm). **d** Measured transmission, *A*_40_/*A*_2_, as a function of the amplitude of the input signal, *A*_2_. **e** Measured cross-correlation of *θ*_2_(*t*) and *θ*_40_(*t*) as a function of the amplitude of the input signal, *A*_2_. **f** Evolution of the pulse velocity *c* as a function of its amplitude. The gray region in **b** and **d**–**f** highlights the amplitude gap as predicted by the continuum model. The error bars in **f** show the 95% confident interval of the measured velocities and amplitudes of solitons in experiments. The corresponding error bars for simulation results are too small to show

Equation (6) indicates that in our system the width of the amplitude gap can be controlled by changing *K*_*s*_, *K*_*θ*_, and *α* (see Supplementary Fig. 14). More excitingly, the tunability and functionality of the proposed mechanical metamaterial can be further enhanced by breaking the symmetry in each rigid cross to alter the coupling strength between the two polarizational components. In our system this is achieved by shifting neighboring horizontal hinges by *a tan φ*_0_ in vertical direction (see Fig. 2a and Supplementary Movie 4). While for the chain with all horizontal hinges aligned (for which *φ*_0_ = 0°) the energy cost to rotate any unit in clockwise and counter-clockwise directions is identical, the hinges shifting (i.e., *φ*_0_ ≠ 0°) introduces a disparity between the two directions of rotation. Under compression in longitudinal direction, for all units of the shifted chain with the left hinge higher than the right one it is energetically more favorable to rotate in clockwise direction, while for the ones with a lower left hinge rotations in counter-clockwise direction are preferred (Fig. 2a and Supplementary Fig. 6). By extending our analytical model to units with *φ*_0_ ≠ 0° and assuming for each unit the positive direction of rotation to be the one that is naturally induced by compression, we find that such disparity introduced by the asymmetry is reflected in the amplitude gap (see Supplementary Note 5). For the aligned chain (i.e., for *φ*_0_ = 0°) the upper (*A*_upper_) and lower (*A*_lower_) limits of the amplitude gap are identical in magnitude (i.e., *A*_lower_ = −*A*_upper_, so that in Fig. 1c, d we only show *A*_upper_). By contrast, as a result of the bias introduced by the hinges shifting, when *φ*_0_ increases, $\left| {A_{{\mathrm{lower}}}} \right|$ and *A*_upper_ become larger and smaller, respectively (see Supplementary Note 5). We also find that a critical angle $\varphi _{\mathrm{0}}^{cr}$ exists at which *A*_upper_ vanishes. In structures with $\varphi _0 > \varphi _{\mathrm{0}}^{cr}$ all solitons that induce an energetically favorable rotation at the *i*-th pair of crosses can propagate through the system, regardless of their magnitude. The validity of our analysis is confirmed by experiments and numerical simulations conducted on a chain comprising 2 × 50 and 2 × 150 crosses characterized by *φ*_0_ = 5°, respectively. For such system our continuum model predicts *A*_upper_ = 0° and *A*_lower_ = −20.91°. In agreement with this analytical prediction, the amplitude transmission ratio and signal shape cross-correlation between the input, *θ*_2_(*t*), and the output, *θ*_40_(*t*), measured in both experiments and discrete simulations significantly drop when the input amplitude falls inside the gap (Fig. 2d, e). Moreover, the numerical and experimental data also closely match the amplitude-velocity relation predicted by our continuum model (Fig. 2f), confirming that the hinges shifting induces an asymmetric gap.

### Functional devices based on amplitude gaps

**Fig. 3:**
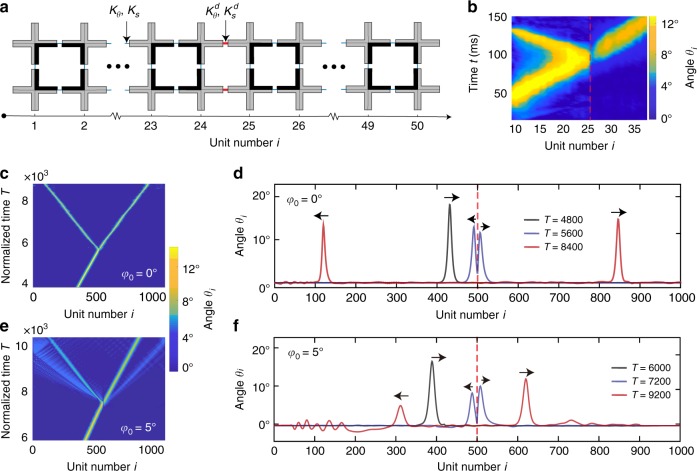
Soliton splitter. **a** Schematics of our soliton splitter. A pair of stiffer hinges (with stiffness $K_s^d$ and $K_\theta ^d$) is introduced to connect the 24th and the 25th pairs of crosses. **b** Rotation of the pairs of crosses during the propagation of the pulse, as recorded with our high-speed camera. The location of the stiff pair of hinges is indicated by the dashed red line. **c**, **d** Simulations corresponding to the experiments shown in **b**. The numerical analysis are conducted on a 2 × 1000 chain with symmetric crosses characterized by *φ*_0_ = 0° and a pair of stiffer hinges placed between the 500th and the 501st units. **e**, **f** Numerical results for a 2 × 1000 chain with asymmetric crosses characterized by *φ*_0_ = 5° and a pair of stiffer hinges placed between the 500th and the 501st units

Having discovered the existence of amplitude gaps in our system, we next focus on how such effect can be exploited to design new functional devices to control mechanical signals, and, therefore, to provide new opportunities for phononic computing and mechanical logic. In analogy to beam splitters^24^, which are widely used in photonics to split an incident light beam into two or more beams, we start by taking advantage of amplitude gaps to design a soliton splitter—a device capable of splitting an incoming elastic vector soliton into a transmitted and a reflected ones. Remarkably, this can be achieved by simply introducing a pair of stiffer hinges within an aligned chain with *φ*_0_ = 0°. To demonstrate the concept we take our 2 × 50 sample with *φ*_0_ = 0° and introduce two stiffer hinges (made of polyester sheets with thickness $t_h^d$ = 0.635 mm) to connect the 24th and the 25th pairs of units (Fig. 3a). We find that, if the amplitude of the input signal is large enough to be outside the amplitude gap, the excited solitary wave is split into two pulses by the pair of stiffer hinges (see Fig. 3b). To better understand the nature of the transmitted and reflected waves, we integrate Eq. (1) to simulate the response of a chain comprising 1000 units with *φ*_0_ = 0 and a pair of stiffer hinges (with stiffness $K_s^d$ and $K_\theta ^d$) connecting the 500th and 501st rigid crosses. The numerical results for a chain with $K_s^d{\mathrm{/}}K_s$ = $K_\theta ^d{\mathrm{/}}K_\theta$ = 30 are shown in Fig. 3c, d. We find that the pair of stiffer hinges split the incoming soliton into two pulses that propagate with stable shape and constant velocity and that no trains of solitons are generated. As for the radiation of linear waves, we find that the interaction between the incoming solitary wave and the stiffer pair of hinges generates only translational vibrations, since the frequency content of our solitons overlaps with a low-frequency band gap for rotation. However, we estimate that only 4% of the energy carried by the incoming soliton is finally transferred to translational linear vibrations (see Supplementary Notes 8–10). As such, these results clearly indicate that our simple structure acts as a splitter for solitons. Note that this behavior is remarkably different from that previously observed in unloaded granular chains, where heterogeneities have been found to split the propagating solitary wave into trains of solitons and to generate stress oscillations localized near the impurities^25^. Such difference is due to the presence of the amplitude gap, which in our mechanical metamaterial prevents fragmentation of the propagating pulse by suppressing the propagation of small amplitude solitons. To demonstrate this important point, we simulate the response of a 2 × 1000 chain with *φ*_0_ = 5° and a pair of stiffer hinges in the middle. Note that this structures enables the propagation of solitary waves of any amplitude if they induce energetically favorable rotations (since *A*_upper_ = 0° and there is no amplitude gap for such waves). We find that, when such waves are excited and hit the pair of stiffer hinges, trains of pulses are generated (see Fig. 3e, f), confirming the important role played by the amplitude gap. Finally, our numerical results also indicate that our soliton splitter is a robust device, since the ratio between the energy carried by the transmitted and reflected solitons only depends on the ratio $K_s^d{\mathrm{/}}K_s$ = $K_\theta ^d{\mathrm{/}}K_\theta$ and not on the amplitude of the input signal, with the amount of reflected energy monotonically increasing with the stiffness ratio (see Supplementary Fig. 23).

**Fig. 4:**
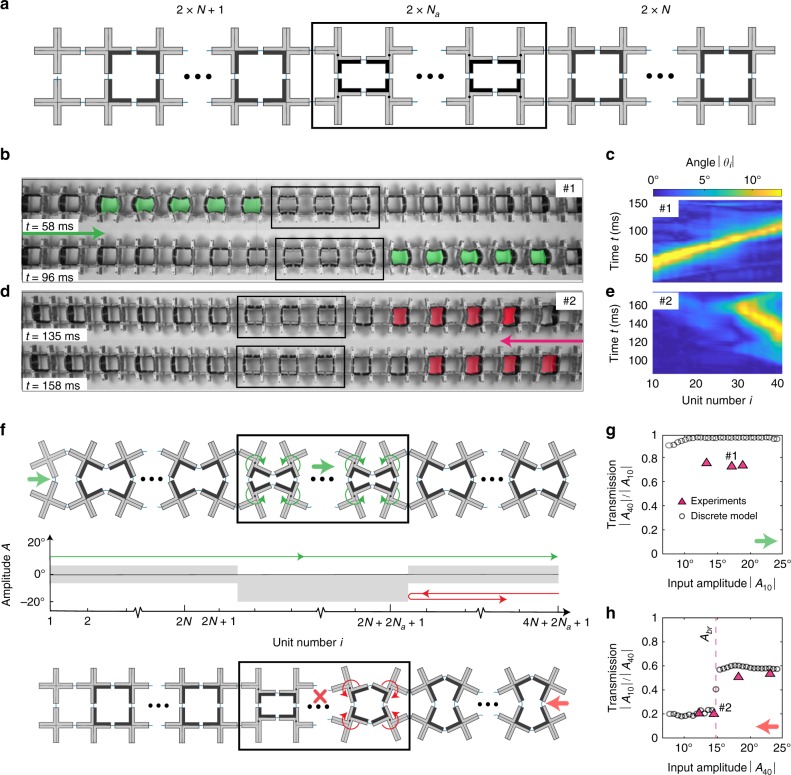
Mechanical diode. **a** Schematics of our mechanical diode. **b** Optical images showing the propagation of a solitary wave excited at the left end of the chain. **c** Rotation of the pairs of crosses induced by a pulse excited at the left of the chain. **d** Optical images showing the propagation of a solitary wave excited at the right end of the chain. **e** Rotation of the pairs of crosses induced by a pulse excited at the right of the chain. **f** Schematic highlighting the working principles of our mechanical diode. **g** Measured transmission, $\left| {A_{40}{\mathrm{/}}A_{10}} \right|$, as a function of the input amplitude, $\left| {A_{10}} \right|$, for pulses excited at the left end of the chain. **h** Measured transmission, $\left| {A_{10}{\mathrm{/}}A_{40}} \right|$, as a function of the input amplitude, $\left| {A_{40}} \right|$, for pulses excited at the right end of the chain

Further, we design a mechanical diode^26^ for solitary waves—a system that is transparent to solitons incoming from one direction but blocks those propagating in the other one. While such nonreciprocal wave transmission has been previously reported for periodic waves^27–29^, irreversible transition waves^30^ and wave packets^31^, here we extend the concept to solitary wave pulses. To achieve this, we introduce a few pairs of crosses with *φ*_0_ ≠ 0 within a chain with *φ*_0_ = 0°. More specifically, our diode comprises two external sections with 2*N* and (2*N* + 1) pairs of crosses characterized by *φ*_0_ = 0 and a central portion consisting of 2*N*_*a*_ pairs of crosses with *φ*_0_ = 5° (Fig. 4a). Experiments conducted on a sample with *N* = 12, *N*_*a*_ = 3 and the section with 2*N* + 1 units placed on the left show that a pulse initiated at the left end propagates through the entire structure (Fig. 4b, c), while a solitary wave excited at the right end is completely reflected by the boundary between the regions with *φ*_0_ = 0° and 5° (Fig. 4d, e). This remarkable behavior is induced by the asymmetric amplitude gap of the region with *φ*_0_ = 5°. Solitons excited at the left end of the chain induce energetically favorable rotations at the units with *φ*_0_ = 5° and, since there is no amplitude gap for such waves (i.e., $A_{{\mathrm{upper}}}^{\varphi _0 = 5^ \circ }$ = 0), they are able to propagate through the entire chain (Fig. 4f). In contrast, solitons initiated at the right end of the chain result in energetically unfavorable rotations for the crosses with *φ*_0_ = 5° and, since $A_{{\mathrm{lower}}}^{\varphi _0 = 5^ \circ } \ll A_{{\mathrm{lower}}}^{\varphi _0 = 0^ \circ }$, they are almost completely blocked by the boundary between aligned and shifted crosses (Fig. 4f). To explore the performance of our mechanical diode, we apply different input signals at the left and right end of the chain in both experiments and discrete simulations. We find that for all pulses with amplitude larger than $A_{{\mathrm{upper}}}^{\varphi _0 = 0} = 6.55^ \circ$ initiated at the left end of the system the transmission, $\left| {A_{40}} \right|/\left| {A_{10}} \right|$, approaches unity (Fig. 4g). Differently, when the excitation is applied at the right end of the chain, the transmission, $\left| {A_{10}} \right|{\mathrm{/}}\left| {A_{40}} \right|$, is close to zero even if the amplitude of the input signal is outside the gap of the region with *φ*_0_ = 0 (i.e., $\left| {A_{40}} \right| > A_{{\mathrm{upper}}}^{\varphi _0 = 0}$). However, as typically observed in electronic and thermal diodes^32^, if the amplitude of the pulses becomes too large, the diode experiences a condition known as breakdown. As a result, solitary waves with amplitude larger than *A*_*br*_ ≈ 15° propagate through the diode (i.e., if $\left| {A_{40}} \right| > A_{br}$ ≈ 15°, then $\left| {A_{10}} \right|{\mathrm{/}}\left| {A_{40}} \right|$ ~ 0.6—see Supplementary Note 11 for a detailed numerical study on the dependency of *A*_*br*_ on *φ*_0_ and *N*_*a*_).

## Discussion

In this study, we have experimentally observed, numerically simulated, and mathematically analyzed the existence of amplitude gaps for elastic vector solitons in highly deformable mechanical metamaterials consisting of rigid units and elastic hinges. First, we have shown that such amplitude gaps can be tuned by altering both the structural parameters and the symmetry of the crosses. Then, we have demonstrated that amplitude gaps can be exploited to design clean splitters and diodes for highly nonlinear solitary waves. In recent years, many strategies have been proposed to manipulate the propagation of elastic waves^29,33^, enabling a wide range of applications such as spatial guiding^34–36^, frequency filtering^37–39^, noise/impact mitigation^40,41^, and non-reciprocal transmission^14,42^. However, the vast majority of devices focus on small-amplitude vibrations and take advantage of spectral gaps in frequency^29,33,43^. As such, our study on amplitude gaps for highly nonlinear solitary waves adds a whole new dimension to our ability to design structures and materials with tailored dynamic behavior and open avenues for potential technological breakthroughs.

## Methods

### Summary of Supplementary Note

Details on fabrication are provided in Supplementary Note 1; on experiments in Supplementary Note 2; on the discrete model in Supplementary Note 3; on the continuum model in Supplementary Note 4; on amplitude gaps for solitons in Supplementary Note 5; on the solution for the aligned chain in Supplementary Note 6; on solitons excited by pulling in Supplementary Note 7; on energy carried by solitons in Supplementary Note 8 and on the dispersion relation in Supplementary Note 9. Furthermore, additional results for the splitter and the diode are shown in Supplementary Notes 10 and 11, respectively.

### Code availability

Matlab code for numerical simulations are included in the Supplementary Files.

## Electronic supplementary material

Supplementary Information

Peer Review File

Description of Additional Supplementary Files

Supplementary Movie 1

Supplementary Movie 2

Supplementary Movie 3

Supplementary Movie 4

Supplementary Dataset 1

Supplementary Dataset 2

Supplementary Dataset 3

Supplementary Dataset 4

## Data Availability

The authors declare that data supporting the findings of this study are included within the paper and its Supplementary Information files or are available from the corresponding author upon reasonable request
