# Supplementary material for: Metamaterials with amplitude gaps for elastic solitons
Source: Nat Commun. 2018 Aug 24;9:3410. doi: 10.1038/s41467-018-05908-9 (PMC6109112; doi:10.1038/s41467-018-05908-9)
Supplement: Supplementary file 1 — Supplementary Information [file 41467_2018_5908_MOESM1_ESM.pdf]

Supplementary Materials for Metamaterials with Amplitude Gaps for Elastic Solitons

Deng et al

Supplementary Note 1: Fabrication

Our system consists of a long chain of 2×50 crosses made of LEGO bricks and connected by thin and flexible hinges made of plastic shims. Two different samples are built: one in which the horizontal hinges are all aligned (see Supplementary Figure 1 and Supplementary Movie 1) and another one in which neighboring horizontal hinges are shifted in vertical direction (see Supplementary Figure 2 and Supplementary Movie 4). In the following we will refer to the former as the aligned chain and to the latter as the shifted chain.

In the aligned chain each cross-shaped unit is realized using four brackets 2×2-2×2 (LEGO part 3956), as shown in Supplementary Figure 1. Differently, the cross-shaped units in the shifted sample are realized using three brackets 2×2-2×2 (LEGO part 3956) and two plates 2×2 (LEGO part 3022), as shown in Supplementary Figure 2. Note that in both samples identical bricks of different colors (black and gray) are used to facilitate visualization of the propagating pulses.

In both samples the hinges are realized by laser cutting the octagonal shape shown in Supplementary Figure 1a and Supplementary Figure 2a out of polyester plastic sheets (Artus Corporation, NJ - 0.005", Blue) with thickness $t_h = 0.127$ mm, Young's modulus $E = 4.33$ GPa and Poisson's ratio $\nu = 0.4$. The size of the octagonal shape is chosen to leave hinges of length $l_h = 4$ mm between the cross-shaped rigid units. Note that eight circular holes are incorporated into each hinge. They fit into the LEGO knobs and enable us to fix the hinges between the interlocking LEGO bricks (see Supplementary Figure 1b and Supplementary Figure 2b).

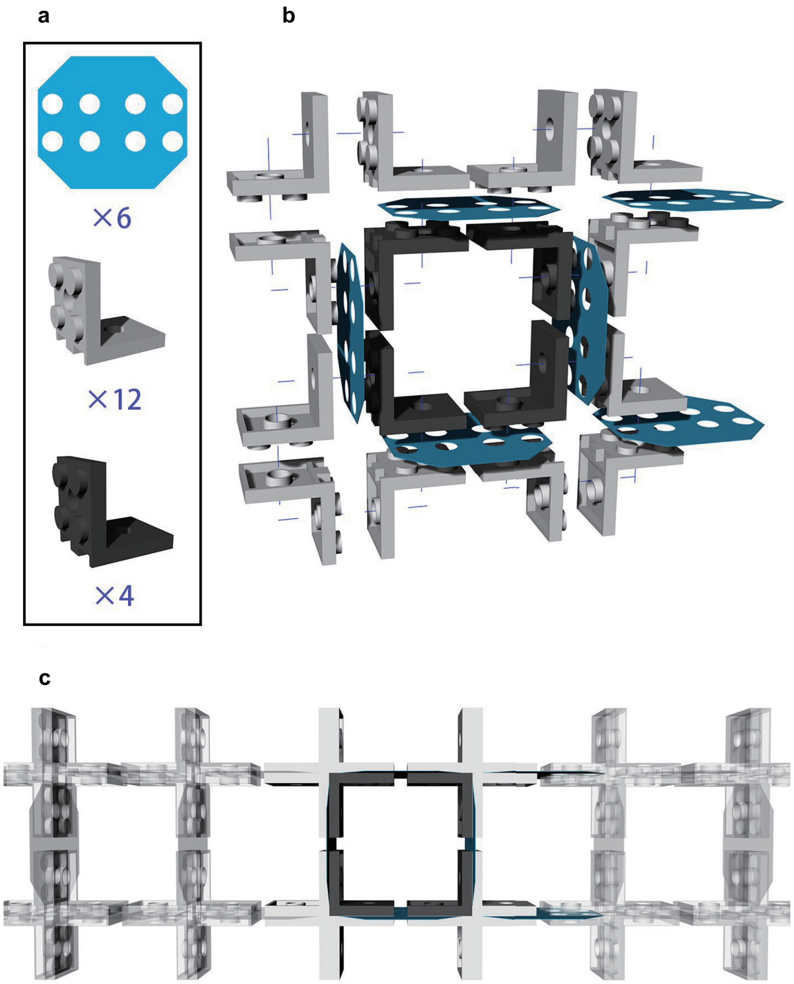

Supplementary Figure 1: Fabrication of the aligned chain. (a) Parts used to fabricate the unit cell. (b) Exploded view of the two pairs of crosses. (c) The chain is realized by putting together a number of unit cells.

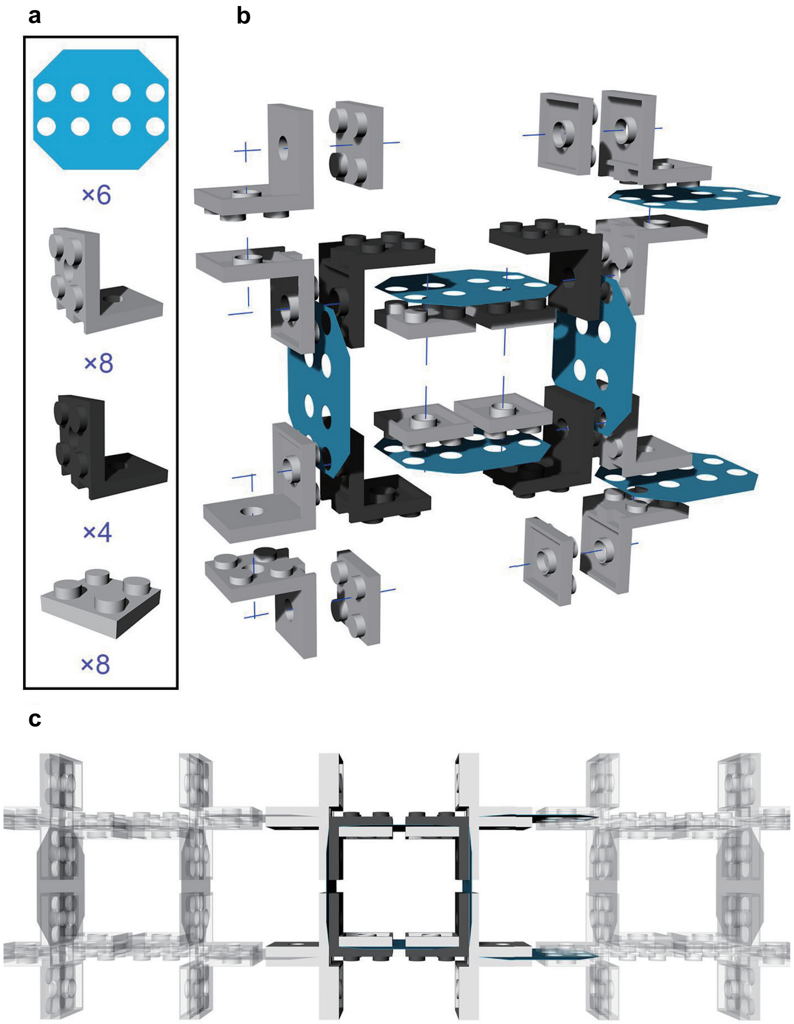

Supplementary Figure 2: Fabrication of the shifted chain. (a) Parts used to fabricate the unit cell. (b) Exploded view of two pairs of crosses. (c) The chain is realized by putting together a number of unit cells.

Supplementary Note 2: Testing

To investigate the propagation of pulses in our samples, we place the chain on a smooth horizontal surface (see Supplementary Figure 3a-b), while supporting each rigid unit with pins to minimize the effect of friction (see Supplementary Figure 3g). We use an impactor excited by a pendulum to hit the mid point at the left end of the chain (see Supplementary Figure 3c-f and Supplementary Movie 2). Different input signals are applied to the chain by varying both the strength of the pulse (controlled by the initial height of the striking pendulum, h_{input} - see Supplementary Figure 3d) and the amplitude of the pulse (controlled by the distance traveled by the impactor, u_{input} - see Supplementary Figure 3c). We find that all of them initiate simultaneous rotation and displacement of the rigid units, with each pair of crosses sharing the same displacement and rotating by the same amount, but in opposite directions (i.e. if the top unit rotates by a certain amount in clockwise direction, then the bottom one rotates by the same amount in counter-clockwise direction, and vice versa). To monitor the displacement, u_i, and rotation, θ_i, of i-th pair of crosses along the chain as the pulses propagate, we use a high speed camera (SONY RX100V) recording at 480 fps and track four markers placed on the external arms of each pair of crosses (see Supplementary Figure 4) via digital image correlation analysis [1]. More specifically, the longitudinal displacement u_i and rotation θ_i of the i-th pair of rigid units is obtained as

$$
\begin{aligned}
u_i(t) &= \frac{1}{2}\sum_{\gamma=1,2}\left[x_i^{(\gamma)}(t) - x_i^{(\gamma)}(0)\right] \\
\theta_i(t) &= \frac{1}{2}\sum_{\gamma=1,2}(-1)^{i+\gamma}\arcsin\left[\frac{\left(x_i^{(\gamma+2)}(t) - x_i^{(\gamma+2)}(0)\right) - \left(x_i^{(\gamma)}(t) - x_i^{(\gamma)}(0)\right)}{\sqrt{\left(x_i^{(\gamma+2)}(0) - x_i^{(\gamma)}(0)\right)^2 + \left(y_i^{(\gamma+2)}(0) - y_i^{(\gamma)}(0)\right)^2}}\right]
\end{aligned}
\tag{S1}
$$

where $\left(x_i^{(\gamma)}(t),\, y_i^{(\gamma)}(t)\right)$ and $\left(x_i^{(\gamma)}(0),\, y_i^{(\gamma)}(0)\right)$ are the coordinates of the γ-th marker placed on the i-th pair of rigid units at time t and that time $t = 0$ (i.e. before the impact), respectively.

Finally, we note that, while for the aligned chain the energy cost to rotate any unit in clockwise and counter-clockwise directions is identical, for the shifted chain there is a disparity between the two directions of rotation. Under compression in the longitudinal direction, for all units of the shifted chain with the left hinge higher than the right one, it is energetically more favorable to rotate in the clockwise direction, while for the ones with a lower left hinge, rotations in counter-clockwise direction are preferred. In our experiments, we can excite any cross of the shifted chain in either direction of rotation by changing the type of unit placed at the left end of the structure. To excite energetically favorable rotations (i.e. to rotate all crosses with the left hinge higher than the right one in clockwise direction and all crosses with the right hinge higher than the left one in counter-clockwise direction), we place a pair of crosses with $\varphi_0 = 0$ at the left end of the chain (see Supplementary Figure 5a). Energetically unfavorable rotations

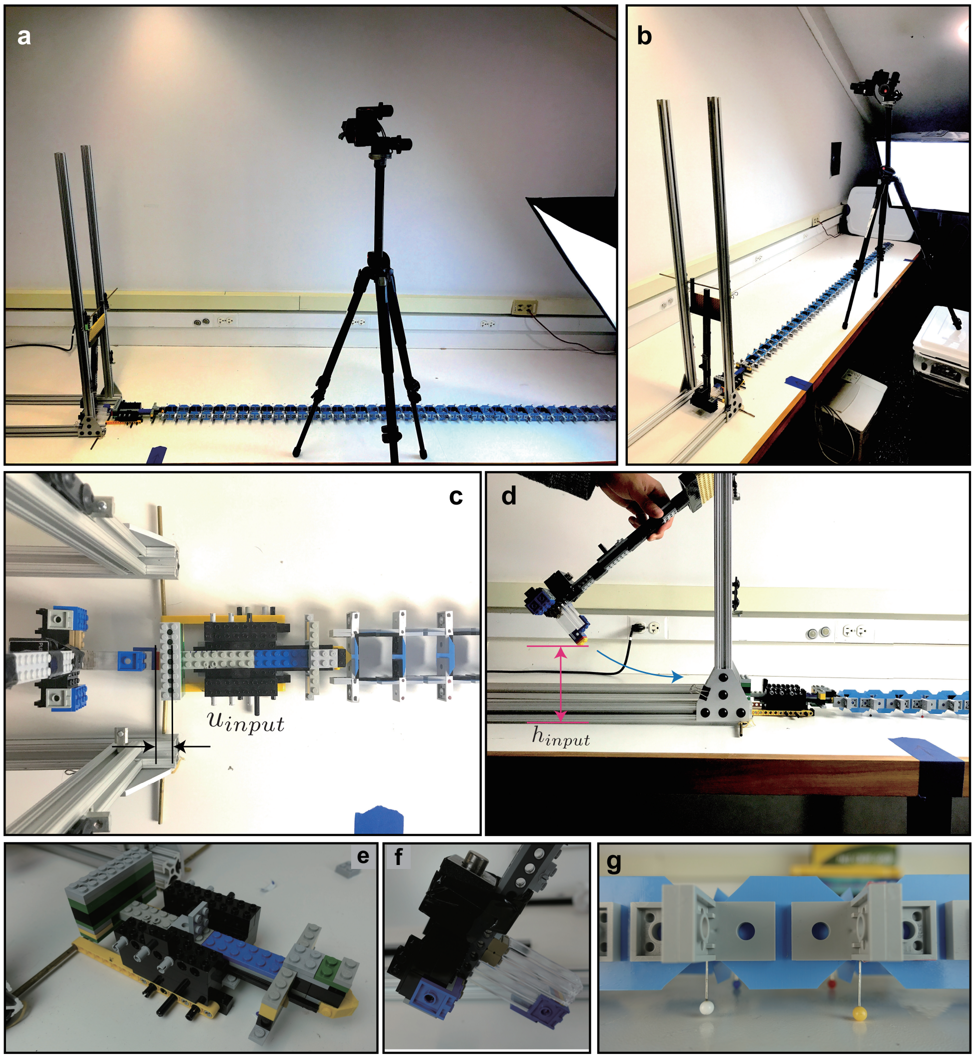

Supplementary Figure 3: Experimental setup. (a)-(b) Pictures of our experimental setup showing the sample, the lamp used to illuminate it, the pendulum and the impactor used to excite the pulses and the camera used to monitor the propagation of the pulses. (c) Top-view of the pendulum and the impactor. (d) Side-view of the pendulum and the impactor. (e) Close-up view of the impactor. (f) Close-up view of the end of the pendulum. (g) Friction is minimized by supporting each rigid unit with pins.

are excited by placing a pair of units with $\varphi_0 \neq 0$ at the left end of the chain (see Supplementary Figure 5b).

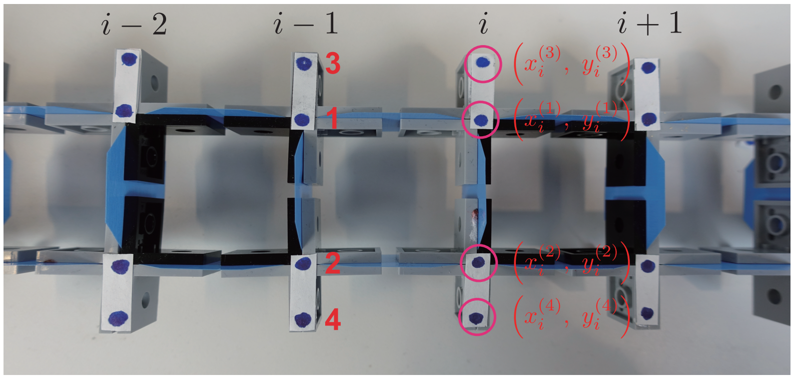

Supplementary Figure 4: Digital image correlation analysis. For each pair of rigid crosses four markers (blue dots) are tracked.

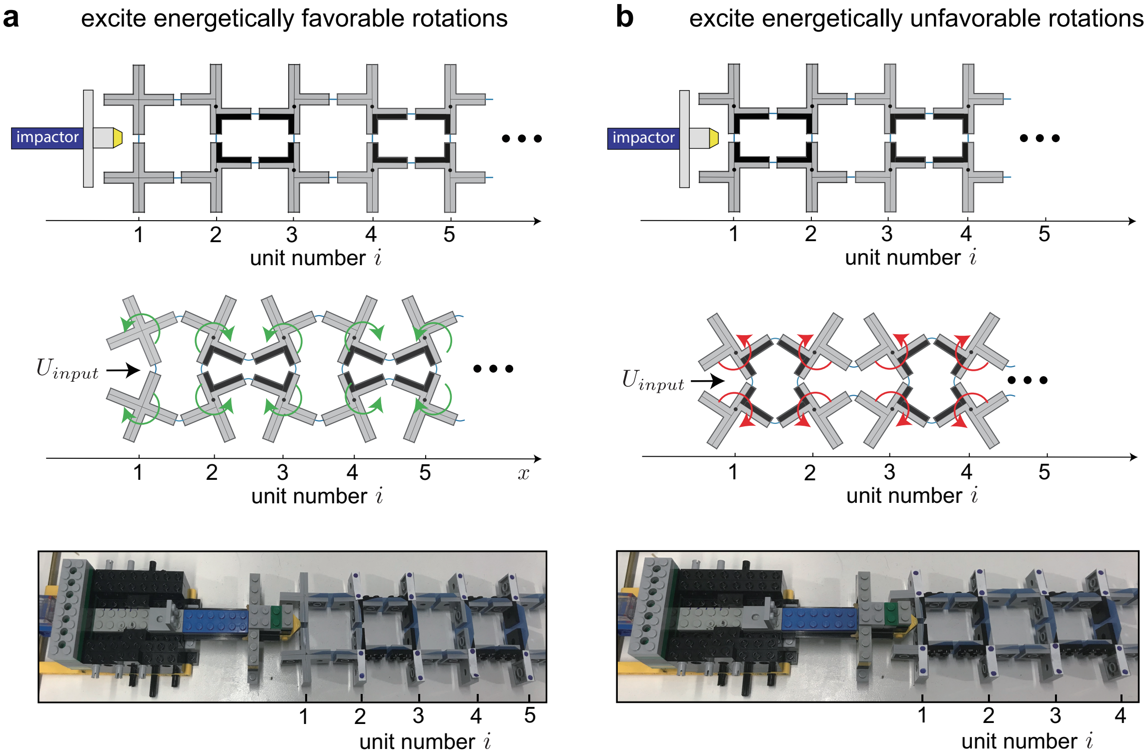

Supplementary Figure 5: Exciting different rotations in the shifted chain. (a) Energetically favorable rotations are excited by placing a pair of crosses with $\varphi_0 = 0$ at the left end of the chain. (b) Energetically unfavorable rotations are excited by placing a pair of crosses with $\varphi_0 \neq 0$ at the left end of the chain.

Supplementary Note 3: Discrete model

Our system consists of a long chain of 2×N crosses with center-to-center distance a connected by thin and flexible hinges (see Supplementary Figure 6). In our model we focus on the most general case in which neighboring horizontal hinges are shifted in vertical direction by $a \tan \varphi_0$ (see Supplementary Figure 6-a). Note that the response of the configuration with all horizontal hinges aligned (see Supplementary Figure 6-b) can be then simply obtained by setting $\varphi_0 = 0$.

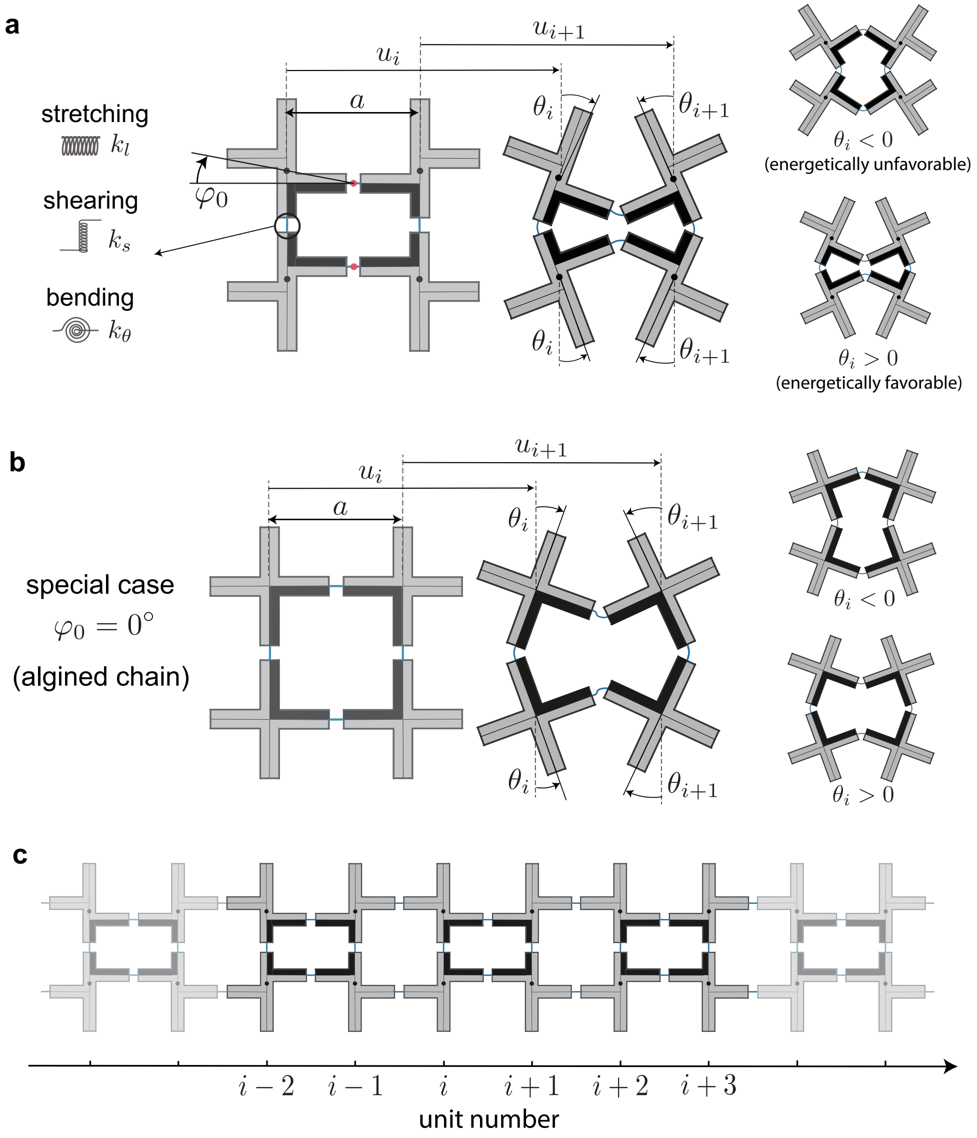

Supplementary Figure 6: Schematics of the system. (a) Configuration in which neighboring horizontal hinges are shifted in vertical direction by $a \sin \varphi_0$. (b) Configuration in the horizontal hinges are all aligned. (c) Chain comprising 2×10 cross-shaped rigid units.

Since in this work we focus on the propagation of longitudinal nonlinear waves along the chain, we assign two degrees of freedom to each rigid cross: the longitudinal displacement u and the rotation in the $x-y$ plane θ. Moreover, guided by our experiments, we assume that each pair of crosses shares the same displacement and rotates by the same amount, but in opposite directions (i.e. if the top cross rotates by a certain amount in clockwise direction, then the bottom one rotates by the same amount in counter-clockwise direction, and vice versa). As such, two degrees of freedom are assigned to the i-th pair of crosses: the longitudinal displacement u_i and the rotation θ_i (see Supplementary Figure 6a and b). Moreover, to facilitate the analysis, we define the positive direction of rotation alternatively for neighboring units along the x-axis. Specifically, for each cross we assume the energetically favorable direction of rotation to be the positive one. As such, for the i-th top unit (for which the left horizontal hinge is higher than the right one - see Supplementary Figure 6a) a clockwise rotation is positive, while for the $i-1$-th and $i+1$-th top ones (for which the left horizontal hinges are lower than the right ones - see Supplementary Figure 6a), counterclockwise rotations are considered positive.

As for the hinges, we model them using a combination of three linear springs: (i) their stretching is captured by a spring with stiffness k_l; (ii) their shearing is governed by a spring with stiffness k_s; (iii) their bending is captured by a torsional spring with stiffness k_θ (see Supplementary Figure 6a).

Under these assumptions, the equations of motion for the i-th pair of crosses are given by

$$\begin{aligned}
m\ddot{u}_i =& k_l\left[u_{i+1}-2u_i+u_{i-1}-\frac{a}{2\cos\varphi_0}\left(\cos(\theta_{i+1}+\varphi_0)-\cos(\theta_{i-1}+\varphi_0)\right)\right],\\
J\ddot{\theta}_i =& -k_\theta(\theta_{i+1}+4\theta_i+\theta_{i-1})+\frac{k_s a^2}{4\cos^2\varphi_0}\cos(\theta_i+\varphi_0)\big[\sin(\theta_{i+1}+\varphi_0)-2\sin(\theta_i+\varphi_0)\\
&+\sin(\theta_{i-1}+\varphi_0)\big]-\frac{k_l a}{2\cos\varphi_0}\sin(\theta_i+\varphi_0)\Big[(u_{i+1}-u_{i-1})\\
&+\frac{a}{2\cos\varphi_0}\big(4\cos(\varphi_0)-\cos(\theta_{i+1}+\varphi_0)-2\cos(\theta_i+\varphi_0)-\cos(\theta_{i-1}+\varphi_0)\big)\Big],
\end{aligned}\tag{S2}$$

where m and J are the mass and moment of inertia of the rigid crosses, respectively.

Next, we introduce the normalized displacement $U_i = u_i/a$, time $T = t\sqrt{k_l/m}$, inertia $\alpha = a/(2\cos\varphi_0)\sqrt{m/J}$ and stiffness ratios $K_\theta = 4k_\theta\cos^2\varphi_0/(k_l a^2)$ and $K_s = k_s/k_l$. Eqs.

(S2) can then be written in dimensionless form as

$$\begin{aligned}\frac{\partial^2 U_i}{\partial T^2} =& U_{i+1} - 2U_i + U_{i-1} - \frac{\cos(\theta_{i+1}+\varphi_0) - \cos(\theta_{i-1}+\varphi_0)}{2\cos\varphi_0},\\ \frac{1}{\alpha^2}\frac{\partial^2 \theta_i}{\partial T^2} =& - K_\theta(\theta_{i+1} + 4\theta_i + \theta_{i-1}) + K_s \cos(\theta_i+\varphi_0)\\ &\times \Big[\sin(\theta_{i+1}+\varphi_0) + \sin(\theta_{i-1}+\varphi_0) - 2\sin(\theta_i+\varphi_0)\Big]\\ &- \sin(\theta_i+\varphi_0)\Big[2\cos(\varphi_0)\,(U_{i+1}-U_{i-1}) + 4\cos(\varphi_0) -\\ &\cos(\theta_{i+1}+\varphi_0) - 2\cos(\theta_i+\varphi_0) - \cos(\theta_{i-1}+\varphi_0)\Big].\end{aligned} \tag{S3}$$

For a chain comprising N pairs of units Eqs. (S3) result in a system of $2N$ coupled differential equations, which can be numerically solved for a given set of initial and boundary conditions. In this study, we use the 4th order Runge-Kutta method (via the Matlab function *ode45*) to numerically solve Eqs. (S3) (the code implemented in MATLAB is available online). Moreover, as initial conditions we set $U = 0$ and $\theta = 0$ for for all pairs of crosses. Finally, we enforce two different sets of boundary conditions:

- First, to compare the numerical predictions to our experimental results and verify the relevance of our discrete model, we apply the experimentally extracted displacement, $u_2(t)$, and rotation, $\theta_2(t)$, signals to the first pair of crosses at the left end of the chain, while implementing free-boundary conditions at the right end;
- Second, to generate the numerical results reported in the main text, as in our experiments, we apply a displacement

  $$U_{input}(T) = b + b\tanh\frac{T - T_0}{w} \tag{S4}$$

  in the longitudinal direction to the mid-point at the left end of the chain (see Supplementary Figure 7). In our simulations we use $b \in [0,\ 0.75]$, $w \in [50,\ 100]$ and choose $T_0 = 400$ to ensure that $U_{input} \to 0$ at $T = 0$. Such applied displacement induces simultaneous translation and rotation of the first pair of crosses, which in turn results in a normalized translation of the excited mid-point equal to $U_1 + 1/2\sin\theta_1$. Finally, is is important to note that, to avoid numerical instabilities, we do not apply $U_{input}(t)$ directly to the mid-point at the left end of the chain, but to a linear spring with stiffness $K_{input} = 1$ connected to it (note that the numerical results are not influenced by the particular value chosen for K_{input}). Therefore, $U_{input}(t)$ results in a longitudinal force

  $$F_{input} = K_{input}\left[U_{input} - U_1 - \frac{1}{2}\sin\theta_1\right], \tag{S5}$$

  and in a moment

  $$M_{input} = \frac{1}{2}F_{input}\cos\theta_1, \tag{S6}$$

applied to the first pair of crosses. As such, the governing discrete equations for the first pair of crosses become

$$\begin{aligned}\frac{\partial^2 U_1}{\partial T^2} =& U_2 - U_1 + \frac{2\cos\varphi_0 - \cos(\theta_1+\varphi_0) - \cos(\theta_2+\varphi_0)}{2\cos\varphi_0} + F_{input},\\ \frac{1}{\alpha^2}\frac{\partial^2 \theta_1}{\partial T^2} =& -K_\theta(\theta_2+3\theta_1) + K_s\cos(\theta_1+\varphi_0)\Big[\sin(\theta_2+\varphi_0) - \sin(\theta_1+\varphi_0)\Big]\\ & -\sin(\theta_1+\varphi_0)\Big[2\cos(\varphi_0)\,(U_2-U_1) + 2\cos(\varphi_0) - \cos(\theta_1+\varphi_0) - \cos(\theta_2+\varphi_0)\Big]\\ & + M_{input},\end{aligned} \tag{S7}$$

while the response of all other crosses is governed by Eqs. (S3).

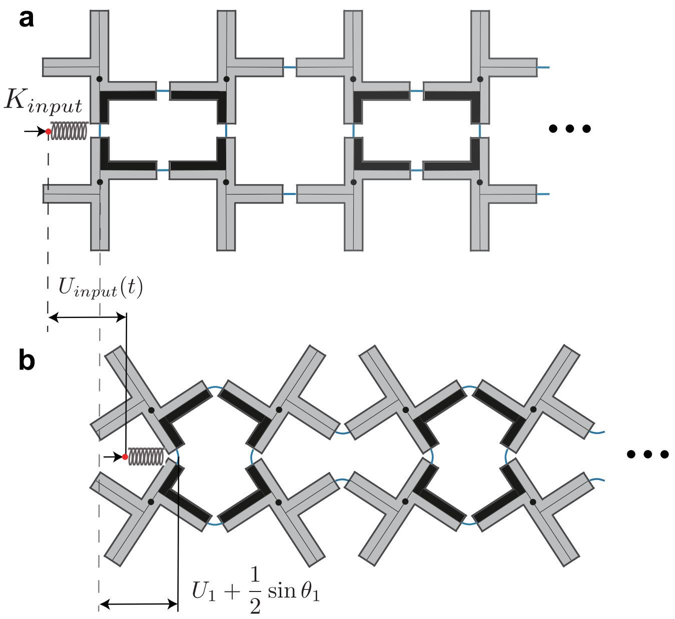

Supplementary Figure 7: Schematics showing how U_{input} is applied in our discrete simulations. (a) A linear spring with stiffness $K_{input} = 1$ is connected to the mid-point at the left end of the chain. (b) U_{input} is applied to this spring.

Estimation of K_θ, K_s and α

To connect the discrete model to our experimental sample, we need to estimate the mass of the cross-shaped units (m), their rotational inertia (J) and the spring stiffnesses (k_l, k_s and k_θ).

Mass m: Since the mass of a single LEGO bracket (LEGO part 3956) is 1.13 g and the mass

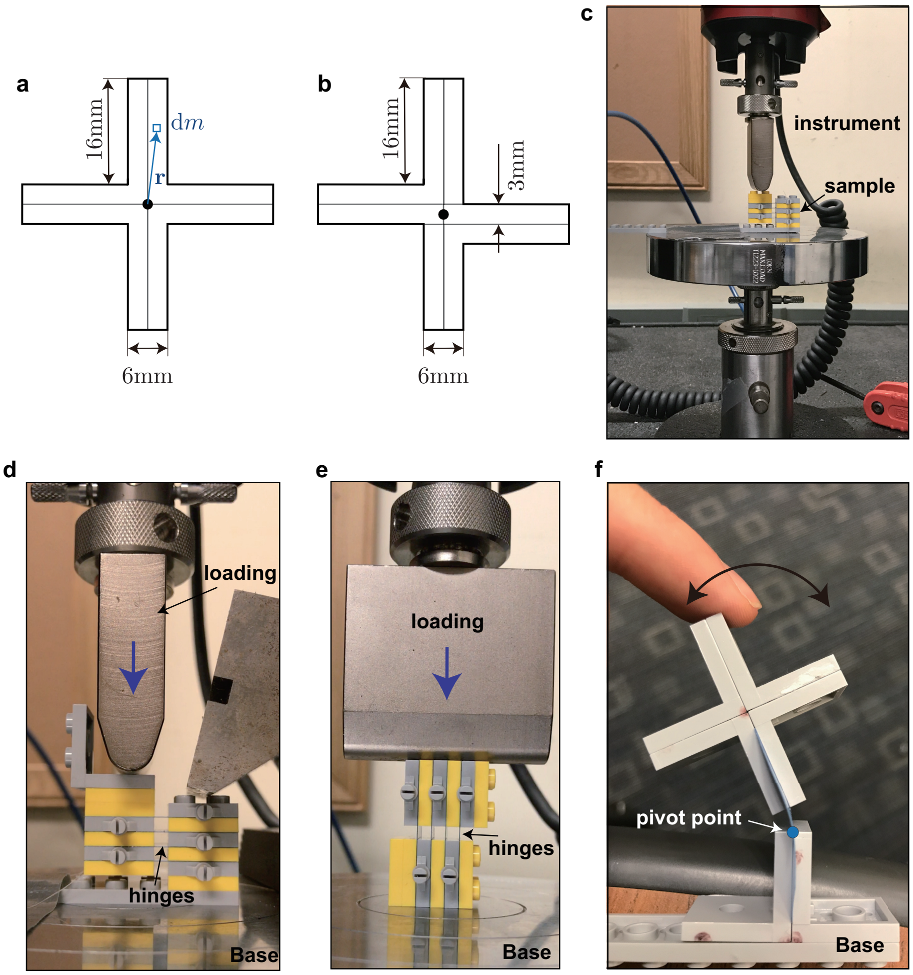

Supplementary Figure 8: (a) Geometry of the rigid unit in our aligned chain. (b) Geometry of the rigid unit in our shifted chain. The back dot represent the center of mass. (c) View of the experimental setup used to estimate k_s and k_l. (d) Picture showing the test conducted to estimate k_s. (e) Picture showing the test conducted to estimate k_l. (f) Picture showing the test conducted to estimate k_θ.

of a single LEGO plate (LEGO part 3022) is 0.60 g, the mass of a rigid cross in our aligned sample is

$$m = 1.13 \times 4 = 4.52\,\mathrm{g}, \tag{S8}$$

and the mass of a rigid cross in our shifted sample is

$$m = 1.13 \times 3 + 0.60 \times 2 = 4.59\,\mathrm{g}. \tag{S9}$$

Rotational inertia J: The rotational inertia J can be calculated from the geometry of the rigid crosses (see Supplementary Figure 8a-b) as

$$J = \int_{area} r^2 \,\mathrm{d}m \tag{S10}$$

where r denotes the distance from the center of mass. We find that for the rigid crosses in the aligned structure $J = 605$ g·mm^2, while for the crosses in the shifted structure $J = 615$ g·mm^2.

Spring stiffness k_s and k_l: To determine the stiffnesses k_s and k_l we build a small structure consisting of four parallel hinges connected at both ends to interlocked LEGO plates (see Fig Supplementary Figure 8c-e). We start by fixing one column of LEGO plates and applying a displacement u_1 to the other one in direction perpendicular to the hinges (see Supplementary Figure 8d), so that the hinges are only subjected to shearing deformations. The stiffness k_s is then obtained from the measured force F_1 as

$$k_s = \frac{F_1}{4u_1} = 1.325\,\frac{\text{N}}{\text{mm}}. \tag{S11}$$

Next, we apply a displacement u_2 to one column of LEGO plates in direction parallel to the hinges (see Supplementary Figure 8e), with u_2 small enough so that the hinges are only compressed axially and do not buckle. The stiffness k_l is then obtained from the measured force F_2 as

$$k_l = \frac{F_2}{4u_2} = 71.69\,\frac{\text{N}}{\text{mm}}. \tag{S12}$$

Spring stiffness k_θ: To determine the stiffness k_θ of the torsional spring we connect an hinge to a fixed base and to a cross made of four LEGO brackets (see Supplementary Figure 8f). We then apply an initial rotation to the systems and let it free to vibrate. k_θ can be obtained from the experimentally measured frequency of vibration f using

$$f = \frac{1}{2\pi}\sqrt{\frac{k_\theta}{J'}} \tag{S13}$$

where $J' = 3000$ g·mm^2 is the rotational inertia of the cross with respect to the pivot point. Since we measure $f = 6.4$ Hz, we find that

$$k_\theta = 4\pi^2 f^2 J' = 4.85\ \ \text{N}\cdot\text{mm}. \tag{S14}$$

Dimensionless parameters K_θ, K_s and α: From the parameters above, we obtain that

- for the aligned structure

$$\alpha = \frac{a}{2}\sqrt{\frac{m}{J}} = 1.815, \quad K_s = \frac{k_s}{k_l} = 0.0185, \quad K_\theta = \frac{4k_\theta}{k_l a^2} = 1.534\times 10^{-4}, \tag{S15}$$

- for the shifted structure

$$\alpha = \frac{a}{2\cos\varphi_0}\sqrt{\frac{m}{J}} = 1.821, \quad K_s = \frac{k_s}{k_l} = 0.0185, \quad K_\theta = \frac{4k_\theta\cos^2\varphi_0}{k_l a^2} = 1.522\times 10^{-4}. \tag{S16}$$

Since the nondimensional parameters for the two different structures are reasonably close, in this study we use

$$\alpha = 1.8, \quad K_s = 0.0185, \quad K_\theta = 1.5 \times 10^{-4}, \tag{S17}$$

for both structures.

Numerical results

To verify the relevance of our discrete model, we apply the experimentally extracted input displacement and rotation to the first pair of crosses and then compare the signal recorded at the 40th unit in both experiment and simulations. In Supplementary Figure 9 we present such comparison for the two experiments shown in Fig. 1c of the main text. We find that the pulse profiles and amplitudes are well captured by the discrete model. When the amplitude of the input signal is large as in experiment $\#1$ (for which $A_2 = \max(\theta_2(t)) = 13°$), our numerical simulations reveal the propagation of a pulse that conserves its amplitude and shape in both degrees of freedom (see Supplementary Figure 9a). Differently, for inputs with small amplitude as in experiment $\#2$ (for which $A_2 = 5°$), the numerical analyses predict an output signal that is severely distorted compared to the input one, in full agreement with our experimental results (see Supplementary Figure 9b).

All of the numerical results reported in the main text are obtained by a displacement $U_{input} = b + b\tanh(T/w)$ in the longitudinal direction to the mid-point at the left end of the chain, as described in Supplementary Note 3: Discrete Model. In Supplementary Figure 10 we show the response of a 2×150 chain with $\varphi_0 = 0°$ excited by an applied displacement characterized by $b = 0.25$ and $w = 50$. The numerical results indicate that the applied input signal excites a pulse with two polarizational components (one rotational and one translational), which propagates with constant amplitude and velocity.

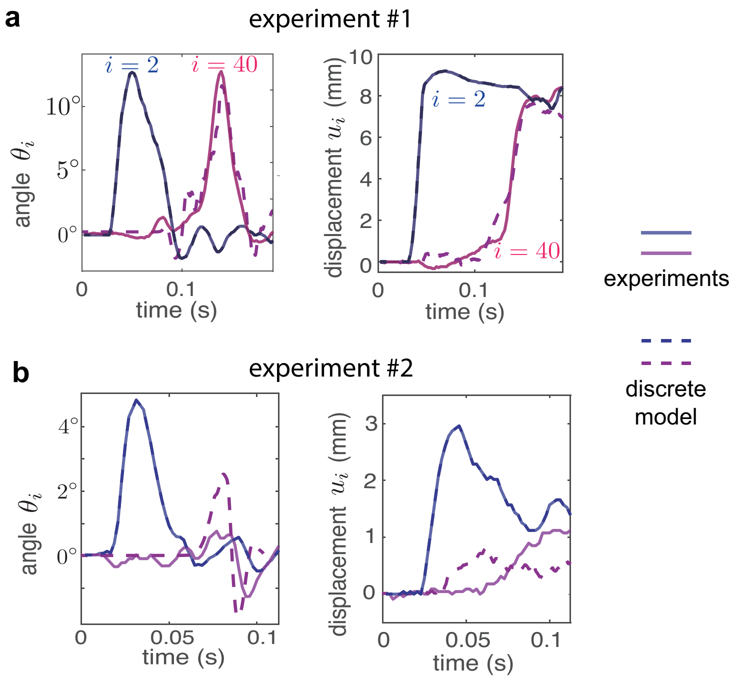

Supplementary Figure 9: Comparison between experimental and numerical results for (a) experiment #1 and (b) experiment #2 shown in Fig. 1c of the main text. Experimental (solid lines) and numerical (dashed lines) signals recorded at the 2nd (blue lines) and 40th (magenta lines) pairs of crosses are reported as a funtion of time. In our numerical simulations, we apply the experimental signal recorded at 2nd pair of crosses as boundary conditions.

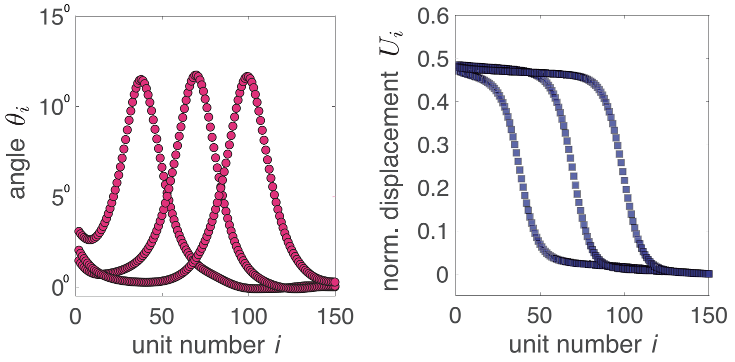

Supplementary Figure 10: Numerical results for a chain comprising 2×150 crosses with $\varphi_0 = 0°$. Rotation (left) and normalized displacement (right) profiles are shown at T = 1050, 1550, and 2050.

Supplementary Note 4: Continuum model

Although Eqs. (S3) can be numerically solved to obtain the response of the system, a deeper insight into the dynamics of the system can be achieved by simplifying the equations to derive analytical solutions. To this end, we introduce two continuous functions $U\left(X,\,T\right)$ and $\theta\left(X,\,T\right)$ that interpolate the displacement and rotation of the i-th pair of crosses located at $x_i = i\,a$ as

$$U\left(X_i,\,T\right) = U_i(T), \quad \theta\left(X_i,\,T\right) = \theta_i(T) \tag{S18}$$

where $X_i = x_i/a = i$. Assuming that the width of the propagating waves is much larger than the unit cell size, the displacement U and rotation θ in correspondence of the $i+1$ and $i-1$-th pairs of crosses can then be expressed using Taylor expansion as

$$\begin{aligned}
U_{i\pm1}(T) &= U\left(X_{i\pm1}, T\right) \approx U\Big|_{X_i,T} \pm \frac{\partial U}{\partial X}\Big|_{X_i,T} + \frac{1}{2}\frac{\partial^2 U}{\partial X^2}\Big|_{X_i,T}\\
\theta_{i\pm1}(T) &= \theta\left(X_{i\pm1}, T\right) \approx \theta\Big|_{X_i,T} \pm \frac{\partial \theta}{\partial X}\Big|_{X_i,T} + \frac{1}{2}\frac{\partial^2 \theta}{\partial X^2}\Big|_{X_i,T}\\
\cos\theta_{i\pm1}(T) &= \cos\Big[\theta\left(X_{i\pm1}, T\right)\Big] \approx \cos\theta\Big|_{X_i,T} \pm \frac{\partial \cos\theta}{\partial X}\Big|_{X_i,T} + \frac{1}{2}\frac{\partial^2 \cos\theta}{\partial X^2}\Big|_{X_i,T}\\
\sin\theta_{i\pm1}(T) &= \sin\Big[\theta\left(X_{i\pm1}, T\right)\Big] \approx \sin\theta\Big|_{X_i,T} \pm \frac{\partial \sin\theta}{\partial X}\Big|_{X_i,T} + \frac{1}{2}\frac{\partial^2 \sin\theta}{\partial X^2}\Big|_{X_i,T}
\end{aligned} \tag{S19}$$

Substitution of Eqs. (S19) into Eqs. (S3) yields

$$\begin{aligned}
\frac{\partial^2 U}{\partial T^2} &= \frac{\partial^2 U}{\partial X^2} - \frac{1}{\cos\varphi_0}\frac{\partial \cos(\theta+\varphi_0)}{\partial X},\\
\frac{1}{\alpha^2}\frac{\partial^2 \theta}{\partial T^2} &= -K_\theta\frac{\partial^2\theta}{\partial X^2} + K_s\cos(\theta+\varphi_0)\frac{\partial^2 \sin(\theta+\varphi_0)}{\partial X^2} + \sin(\theta+\varphi_0)\frac{\partial^2 \cos(\theta+\varphi_0)}{\partial X^2}\\
&\quad - 6K_\theta\theta - 4\sin(\theta+\varphi_0)\Big[\cos\varphi_0\frac{\partial U}{\partial X} + \cos\varphi_0 - \cos(\theta+\varphi_0)\Big],
\end{aligned} \tag{S20}$$

which represent the continuum governing equations of the system. Since these two coupled partial differential equations cannot be solved analytically, guided by our experiments, we further assume that $\theta \sim \varphi_0 \ll 1$, so that

$$\begin{aligned}
\sin(\theta+\varphi_0) &\approx \sin\varphi_0 + \theta\cos\varphi_0 - \frac{\sin\varphi_0}{2}\theta^2 - \frac{\cos\varphi_0}{6}\theta^3,\\
\cos(\theta+\varphi_0) &\approx \cos\varphi_0 - \theta\sin\varphi_0 - \frac{\cos\varphi_0}{2}\theta^2 + \frac{\sin\varphi_0}{6}\theta^3.
\end{aligned} \tag{S21}$$

By substituting Eqs. (S21) into Eqs. (S20) and retaining the nonlinear terms up to third order,

we obtain

$$
\begin{aligned}
\frac{\partial^2 U}{\partial T^2} &= \frac{\partial^2 U}{\partial X^2} + \left[\tan(\varphi_0) + \theta - \frac{\tan(\varphi_0)}{2}\theta^2\right]\frac{\partial \theta}{\partial X},\\
\frac{1}{\alpha^2}\frac{\partial^2 \theta}{\partial T^2} &= (K_s - K_\theta)\frac{\partial^2 \theta}{\partial X^2} - 2\left(2\sin^2(\varphi_0) + 3K_\theta\right)\theta - 3\sin(2\varphi_0)\theta^2 - \frac{7\cos(2\varphi_0)-1}{3}\theta^3\\
&\quad - \left[2\sin(2\varphi_0) + 4\cos^2(\varphi_0)\theta - \sin(2\varphi_0)\theta^2\right]\frac{\partial U}{\partial X}.
\end{aligned}
\tag{S22}
$$

Finally, we introduce the traveling wave coordinate $\zeta = X - cT$, c being the normalized pulse velocity (the dimensional pulse velocity is $ca\sqrt{k/m}$), so that Eqs. (S22) become

$$
\begin{aligned}
\frac{\partial^2 U}{\partial \zeta^2} &= -\frac{1}{1-c^2}\left[\tan\varphi_0\frac{\partial \theta}{\partial \zeta} + \theta\frac{\partial \theta}{\partial \zeta} - \frac{\tan\varphi_0}{2}\theta^2\frac{\partial \theta}{\partial \zeta}\right],\\
\frac{1}{\beta}\frac{\partial^2 \theta}{\partial \zeta^2} &= 2\left(2\sin^2\varphi_0 + 3K_\theta\right)\theta + 3\sin(2\varphi_0)\theta^2 + \frac{7\cos(2\varphi_0)-1}{3}\theta^3\\
&\quad + \left[2\sin(2\varphi_0) + 4\cos^2(\varphi_0)\theta - \sin(2\varphi_0)\theta^2\right]\frac{\partial U}{\partial \zeta},
\end{aligned}
\tag{S23}
$$

where

$$
\beta = \frac{\alpha^2}{\alpha^2(K_s - K_\theta) - c^2}. \tag{S24}
$$

By integrating Eq. (S23)$_1$ with respect to ζ we obtain,

$$
\frac{\partial U}{\partial \zeta} = -\frac{1}{1-c^2}\left[\theta\tan\varphi_0 + \frac{\theta^2}{2} - \frac{\tan(\varphi_0)}{6}\theta^3\right] + C \tag{S25}
$$

where C is the integration constant. Since in this study we focus on the propagation of waves with a finite temporal support and do not consider periodic waves, we require that

$$
\left.\frac{\partial U}{\partial \zeta}\right|_{\zeta\to\infty} = 0, \tag{S26}
$$

from which we obtain $C = 0$. Substitution of Eq. (S25) into Eq. (S23)$_2$ yields

$$
\frac{\partial^2 \theta}{\partial \zeta^2} = C_1\theta + C_2\theta^2 + C_3\theta^3 \tag{S27}
$$

with

$$
\begin{aligned}
C_1 &= 2\beta\left[3K_\theta - \frac{2c^2\sin^2\varphi_0}{1-c^2}\right],\\
C_2 &= -\frac{3\beta c^2}{(1-c^2)}\sin(2\varphi_0),\\
C_3 &= -\frac{\beta c^2}{3(1-c^2)}\left(7\cos(2\varphi_0) - 1\right),
\end{aligned}
\tag{S28}
$$

which is the Klein-Gordon equation with quadratic and cubic nonlinearities [2]. Note that Eq. (S27) differs from the equation recently derived to study the propagation of solitary waves in structures comprising a network of squares connected by thin hinges [3]. While in the previous work only terms up to the second order were considered in the continuum model (see Eq. (8) in [3]), here also the third order terms are included. This enable us to investigate waves with amplitude equal or larger than φ_0.

The analytical solution of Eq. (S27) exists in the form of

$$\theta = \frac{1}{D_1 \pm \ D_2 \cosh\left(\zeta/W\right)} \tag{S29}$$

where D_1, D_2 and W are solution parameters. Eq. (S29) defines a solitary wave with characteristic width W and amplitude

$$A = \theta(\zeta = 0) = \frac{1}{D_1 \pm \ D_2}. \tag{S30}$$

Next, we determine D_1, D_2 and W as a function of the geometry of the system and the pulse velocity c. To this end, we substitute the solution (S29) into Eq. (S27) and find that the latter is identically satisfied only if

$$D_1 = -\frac{C_2}{3C_1}, \quad D_2 = \sqrt{\frac{C_2^2}{9C_1^2} - \frac{C_3}{2C_1}}, \quad \text{and} \quad W = \frac{1}{\sqrt{C_1}}, \tag{S31}$$

where C_1, C_2 and C_3 are defined in Eqs. (S28). Finally, the solution for the displacement U is found by integrating Eq. (S25) with θ given by Eq. (S29),

$$\begin{aligned} U &= \int_{\zeta}^{\infty} \frac{1}{1-c^2} \left[\tan\varphi_0\, \theta(\zeta') + \frac{\theta(\zeta')^2}{2} - \frac{\tan\varphi_0}{6} \theta(\zeta')^3 \right] d\zeta' \\ &= \frac{W}{12(1-c^2)} \left[2 \left(\arctan \frac{D_1 \pm D_2}{\sqrt{D_2^2 - D_1^2}} - \arctan \frac{(D_1 \pm D_2)\tanh(\zeta/W)}{\sqrt{D_2^2 - D_1^2}} \right) \right. \\ &\left(6D_1 (D_2^2 - D_1^2)^{-3/2} + \tan\varphi_0 \frac{D_2^2 - 12D_1^4 - 12D_2^4 + D_1^2(2 + 24D_2^2)}{(D_2^2 - D_1^2)^{5/2}} \right) \\ &+ \frac{6(D_2^2 - D_1^2) + 3D_1 \tan\varphi_0}{(D_1^2 - D_2^2)^2} - D_2 \sinh\frac{\zeta}{W} \left(\frac{\pm 6}{(D_1^2 - D_2^2)(D_1 \mp D_2 \cosh(\zeta/W))} \right. \\ &\left. \left. + \tan\varphi_0 \frac{\mp 4D_1^2 \pm D_2^2 + 3D_1 D_2 \cosh(\zeta/W)}{(D_1^2 - D_2^2)^2 (D_1 \mp D_2 \cosh(\zeta/W))^2} \right) \right] \end{aligned} \tag{S32}$$

As recently found for structures comprising a network of squares connected by thin and highly deformable ligaments [3], Eqs. (S29) and (S32) reveal that our system supports the propagation of elastic vector solitons (i.e. solitary waves with two components - one translational and one rotational - that are coupled together and co-propagate without dispersion).

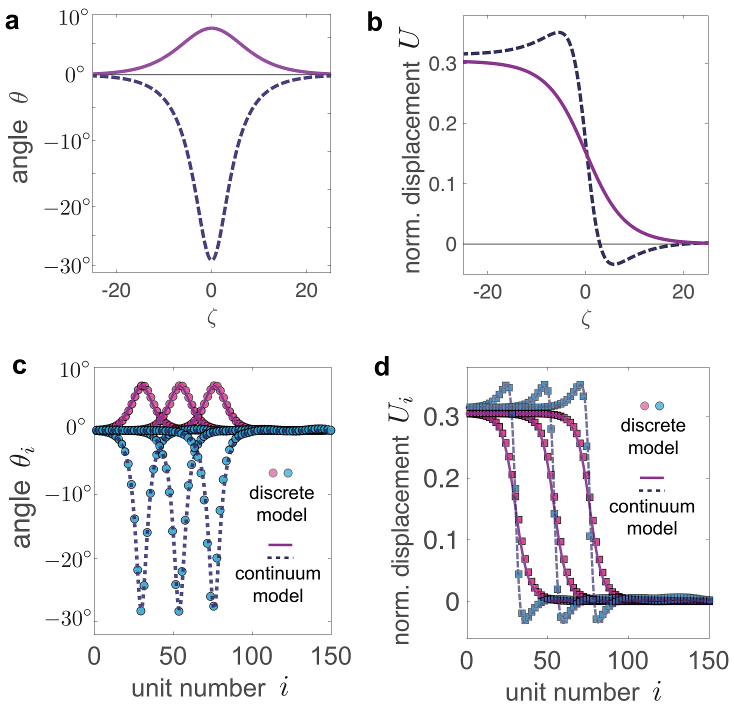

Supplementary Figure 11: (a)-(b) Analytical solution for a structure characterized by $K_s = 0.0185$, $K_\theta = 1.5 \times 10^{-4}$, $\alpha = 1.8$, $c = 0.1$ and $\varphi_0 = 5°$. (c)-(d) Comparison between analytically (lines) and numerically (markers) predicted rotation and normalized displacement profiles at T = 840, 1080, and 1320. Also in this case we assume that $K_s = 0.0185$, $K_\theta = 1.5 \times 10^{-4}$, $\alpha = 1.8$, $c = 0.1$ and $\varphi_0 = 5°$.

In Supplementary Figure 11a-b we report the solution given by Eqs. (S29) and (S32), assuming that $K_s = 0.02$, $K_\theta = 1.5 \times 10^{-4}$, $\alpha = 1.8$, $c = 0.1$ and $\varphi_0 = 5°$. We find that the two vector solitons supported by the system are both characterized by positive values of longitudinal displacement U (i.e. both waves are accompanied by a translation from left to right - see Supplementary Figure 11b). As for the rotational component, one solution is characterized by positive θ and the other one by negative θ (see Supplementary Figure 11a). This means that in one case the top unit of the i-th pair of crosses rotates clockwise, while in the other it rotates counter-clockwise (in both cases neighboring units rotate in opposite directions). It is also important to note that, while the two solutions for U have comparable magnitude, the negative solution for θ (i.e. the one that involves counter-clockwise rotation for the top unit of the i-th pair of crosses) has much larger amplitude than the positive one. Such disparity is due to the asymmetry of the structure with $\varphi_0 \neq 0$, which makes rotation in clockwise direction for the i-th unit (for which the left horizontal hinge is higher than the right one) energetically more favorable. Finally, to verify the validity of our analytical solution, in Supplementary Figure 11c-d we compare the analytical solutions to numerical results obtained by direct integration of the full discrete model (Eqs. (S3)). Note that in this set of simulations we consider a chain with 2×150 crosses and input our theoretical solutions (i.e. Eqs. (S29) and (S32)) as boundary con-

ditions for the first pair of crosses at the left end of the chain, while implementing free-boundary conditions at its right end. We find an excellent agreement between our analytical (lines) and numerical (markers) results, indicating that the assumptions we made, i.e., wave length $\ll$ unit length and $\theta \sim \varphi_0 \ll 1$, are appropriate.

Supplementary Note 5: Amplitude gaps for solitons

Eq. (S29) defines the solitary waves with stable profile that propagate in our system. However, inspection of Eqs. (S29) and (S31) reveals that such waves exist only if:

(i) W is real, yielding

$$C_1 > 0 \tag{S33}$$

since an imaginary W results in a periodic solution, which violates Eq. (S26);

(ii) D_2 is a real number, yielding

$$\frac{C_2^2}{9C_1^2} - \frac{C_3}{2C_1} > 0 \tag{S34}$$

since we want the solution θ to be real;

(iii) the denominator in Eq. (S29) is different from zero, yielding

$$D_1 \pm D_2 \cosh(W\zeta) \neq 0 \quad \forall\, \zeta \tag{S35}$$

since we require the solution θ to be of finite amplitude. Note that, since $D_2 > 0$ (see Eq. (S34)) and $\cosh(W\zeta) \in [1, \infty)$, if we require both solutions of Eq. (S29) to exist, Eq. (S35) can be rewritten as

$$-D_2 < D_1 < D_2. \tag{S36}$$

Differently, if $D_1 > D_2$ or $D_1 < -D_2$, only one of the two solitary waves supported by the system exists. While in this Section we focus on the case where condition (S36) is satisfied (so that both solutions exist), in Supplementary Note 6: Solitons excited by pulling, we investigate the case in which only one solution exists.

By substituting Eqs. (S24) and (S28) into conditions (S33), (S34) and (S36) and assuming that $c < 1$ (note that in all our experiments and simulations $c \in [0.05, 0.5]$) and that $\varphi_0 < 0.7137 = 41^\circ$ (so that $7\cos(2\varphi_0) > 1$), we find that solitary waves exist in our system only if

$$c^2 < \alpha^2 (K_s - K_\theta) \quad \text{and} \quad c^2 < \frac{3K_\theta}{2\sin^2\varphi_0 + 3K_\theta}. \tag{S37}$$

Conditions (S37) clearly show that there is an upper limit for the velocity of the propagating solitary waves, i.e.,

$$c < c_{max} \tag{S38}$$

where

$$c_{max} = \min\left\{ \alpha\sqrt{K_s - K_\theta},\ \sqrt{\frac{3K_\theta}{2\sin^2\varphi_0 + 3K_\theta}} \right\}. \tag{S39}$$

Substitution of condition (S38) into Eq. (S30) yields

$$A > A_{upper} = \left.\frac{1}{D_1 + D_2}\right|_{c=c_{max}} \quad \text{and} \quad A < A_{lower} = \left.\frac{1}{D_1 - D_2}\right|_{c=c_{max}} \tag{S40}$$

indicating that solitary waves can propagate in our system only if their amplitude is larger than A_{upper} and lower that A_{lower}. Therefore, conditions (S40) define an amplitude gap for solitons. By substituting Eqs. (S31) and (S28) into (S40), A_{upper} and A_{lower} can be expressed in terms of structural parameters as,

$$\begin{aligned} A_{upper} &= \frac{2\sqrt{3/\gamma}\sqrt{2\gamma\cos(4\varphi_0) + \cos(2\varphi_0)\left[21K_\theta - (21K_\theta + 8)\gamma\right] + 6\gamma + 3K_\theta(\gamma - 1)} - 6\alpha\sin(2\varphi_0)}{7\cos(2\varphi_0) - 1}, \\ A_{lower} &= \frac{-2\sqrt{3/\gamma}\sqrt{2\gamma\cos(4\varphi_0) + \cos(2\varphi_0)\left[21K_\theta - (21K_\theta + 8)\gamma\right] + 6\gamma + 3K_\theta(\gamma - 1)} - 6\alpha\sin(2\varphi_0)}{7\cos(2\varphi_0) - 1} \end{aligned} \tag{S41}$$

if $\sin\varphi_0 < \sqrt{3K_\theta(1-\gamma)/(2\gamma)}$, or as

$$A_{upper} = 0 \qquad \text{and} \qquad A_{lower} = \frac{-12\sin(2\varphi_0)}{7\cos(2\varphi_0) - 1} \tag{S42}$$

if $\sin\varphi_0 > \sqrt{3K_\theta(1-\gamma)/(2\gamma)}$, with $\gamma = \alpha^2(K_s - K_\theta)$.

In Supplementary Figure 12a-c we report the evolution of A_{upper} (solid purple line) and A_{lower} (solid blue line) as a function of φ_0 for chains characterized by $\alpha = 1.8$ and (K_θ, K_s)=(1.5×10^{-4}, 0.02) (Supplementary Figure 12a), (1.0×10^{-4}, 0.02) (Supplementary Figure 12b) and (1.5×10^{-4}, 0.1) (Supplementary Figure 12c). We find that, while for $\varphi_0 = 0°$ (i.e. when all horizontal hinges are aligned) $A_{lower} = -A_{upper}$, $|A_{lower}|$ and A_{upper} become larger and smaller, respectively, as φ_0 increases. This is because the hinges shifting (i.e. $\varphi_0 \neq 0°$) introduces a disparity between the two directions of rotation. Under compression in the longitudinal direction, for all units of the shifted chain with the left hinge higher than the right one, it is energetically more favorable to rotate in the clockwise direction, while for the ones with a lower left hinge, rotations in the counter-clockwise direction are preferred. As such, pulses that excite energetically favorable rotations can propagate more easily than those inducing energetically unfavorable rotations. We also note that a critical angle

$$\varphi_0^{cr} = \arcsin\sqrt{\frac{3K_\theta(1-\gamma)}{2\gamma}} \tag{S43}$$

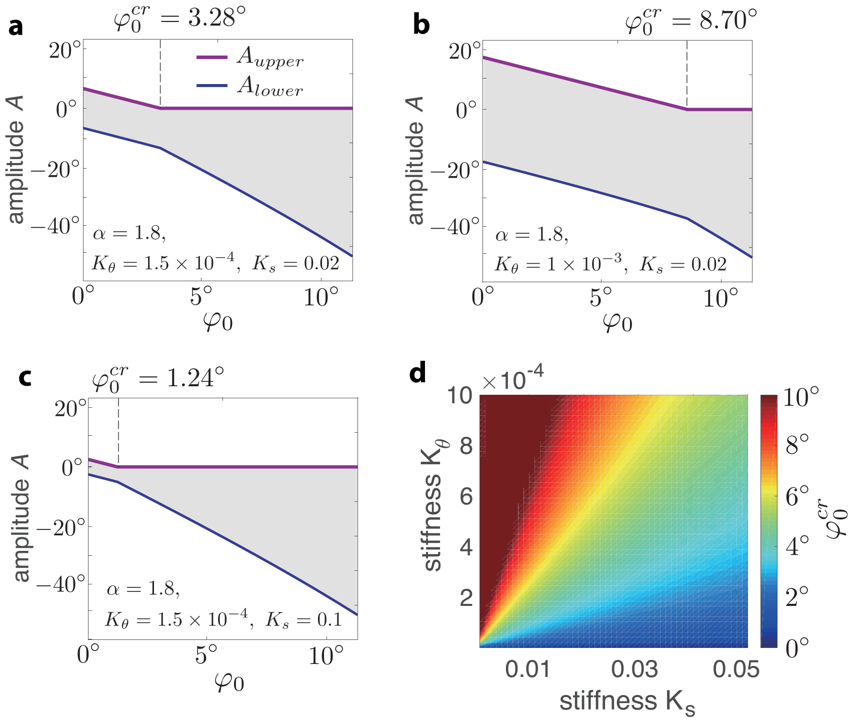

Supplementary Figure 12: (a)-(c) Evolution of the amplitude gap as a function of the angle φ_0 chains characterized by (a) α=1.8, K_θ=1.5 $\times$ 10^{-4} and K_s=0.02; (b) α=1.8, K_θ=1.0 $\times$ 10^{-3} and K_s=0.02; (c) α=1.8, K_θ=1.5 $\times$ 10^{-4} and K_s=0.1. (d) Evolution of φ_0^{cr} as a function of K_s and K_θ, assuming $\alpha = 1.8$.

exists at which A_{upper} eventually vanishes. In structures with $\varphi_0 > \varphi_0^{cr}$ all solitons that induce energetically favorable rotations can propagate through the system, irrespectively of their magnitude. In Supplementary Figure 12d we report the evolution of the angle φ_0^{cr} as a function of K_s and K_θ (assuming $\alpha = 1.8$). We find that φ_0^{cr} increases for larger values of K_θ, while it decreases as K_s becomes larger.

Focusing on the mechanism behind the emergence of the observed amplitude gap for solitons, it is important to note that the propagations of vector solitons require a strong coupling among different polarizations [4, 5]. As such, we expect such amplitude gaps to emerge when there is weak coupling between the two polarizational components. According to Eqs. (S22), the coupling terms in our system are

$$\left[\tan\varphi_0 + \theta - \frac{\tan\varphi_0}{2}\theta^2\right]\frac{\partial\theta}{\partial X} \sim (\varphi_0 + \theta)\,\frac{\partial\theta}{\partial X} \tag{S44}$$

and

$$\left[2\sin(2\varphi_0) + 4\theta\cos^2\varphi_0 - \theta^2\sin(2\varphi_0)\,\right]\frac{\partial U}{\partial X} \sim 4\,(\varphi_0 + \theta)\,\frac{\partial U}{\partial X}. \tag{S45}$$

Eqs. (S44) and (S45) clearly show that if $\varphi_0 = 0$ (i.e. for the aligned structure), the coefficients of both coupling terms are proportional to θ, so that large enough rotations are needed in order

to activate them and enable the propagation of vector solitons. Differently, if $\varphi_0 \neq 0$ (i.e. for the shifted structure), the coefficients are proportional to $\varphi_0 + \theta$ and the strength of the coupling depends on the direction of rotation. The coupling among U and θ is strong when the wave induces an energetically favorable rotation (since φ_0 and θ have the same sign and their effect sums up), resulting in lower values for A_{uppers} as φ_0 increases. By contrast, if the wave induces an energetically unfavorable rotation (i.e. $\theta < 0$), large rotations are required to make the coupling strong enough (i.e. $\theta < 0$ has to go more negative to compensate for the bias induced by the positive φ_0), resulting in large $|A_{lower}|$.

Supplementary Note 6: Solution for the aligned chain

While in Supplementary Note 4: Continuum model and Supplementary Note 5: Amplitude gaps for solitons we derived the analytical solution for the most general case of a system in which neighboring horizontal hinges are shifted vertically by $a \sin \varphi_0$, here we specialized it to a structure in which all horizontal hinges are aligned (i.e. $\varphi_0 = 0$). If $\varphi_0 = 0$, the solution given by Eqs. (S29)-(S32) reduces to,

$$\theta = A \text{ sech}\left(\frac{\zeta}{W}\right), \quad U = \frac{A^2 W}{2(1-c^2)}\left[1 - \tanh\left(\frac{\zeta}{W}\right)\right] \tag{S46}$$

with

$$A = \pm\sqrt{\frac{6K_\theta(1-c^2)}{c^2}} \text{ and } W = \sqrt{\frac{\alpha^2(K_s - K_\theta) - c^2}{6\alpha^2 K_\theta}}. \tag{S47}$$

In Supplementary Figure 13a-b we plot the analytical solution given by Eqs. (S46), assuming $K_s = 0.0185$, $K_\theta = 1.5 \times 10^{-4}$, $\alpha = 1.8$ and $c = 0.1$. We find that the two solutions supported by the system are characterized by identical translational components. The rotational components are also identical, but have opposite direction. Note that the two solutions have the same magnitude because in the aligned chain the units do not have a preferential direction of rotation. Finally, in Supplementary Figure 13c-d, we compare the analytical solution to numerical results obtained by direct integration of the full discrete model (Eqs. (S3)). Note that in this set of simulations we consider a chain with 2×150 crosses and assign the displacement and rotation signals given by Eqs. (S46) to the first pair of units on the left , while keeping free boundary conditions at the right end. Again, we find an excellent agreement between our analytical (lines) and numerical (markers) results.

As for the amplitude gap, if $\varphi_0 = 0$, Eqs. (S41) and (S42) reduce to

$$A_{upper} = -A_{lower} = \sqrt{\frac{6K_\theta}{\alpha^2(K_s - K_\theta)} - 6K_\theta}. \tag{S48}$$

In Supplementary Figure 14 we report the evolution $A_{upper} = -A_{lower}$ as a function of K_s, K_θ and α. The contour plots indicate that A_{upper} can be tuned by varying either K_s or K_θ, while α has a more moderate effect.

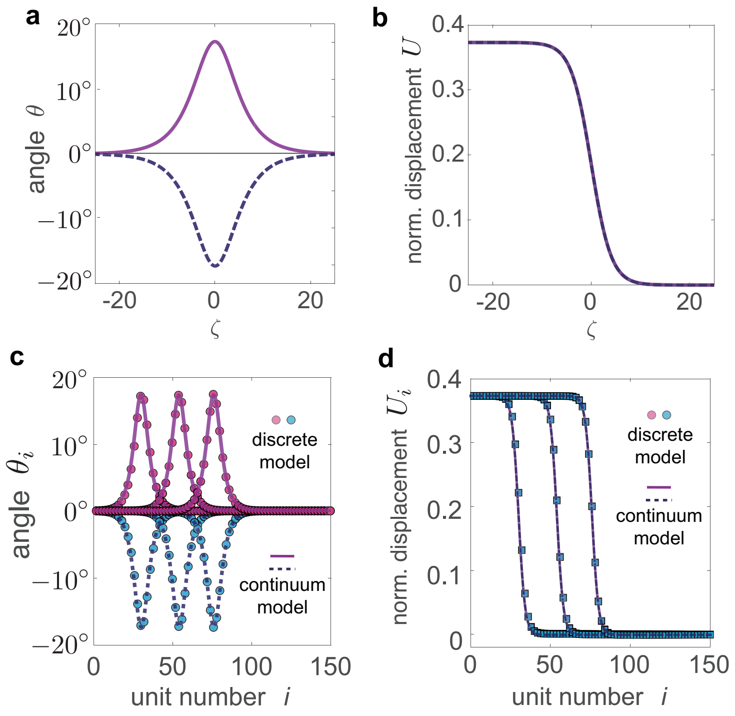

Supplementary Figure 13: (a)-(b) Analytical solution for a structure characterized by $K_s = 0.0185$, $K_\theta = 1.5 \times 10^{-4}$, $\alpha = 1.8$, $c = 0.1$ and $\varphi_0 = 0.0$. (c)-(d) Comparison between analytically (lines) and numerically (markers) predicted rotation and normalized displacement profiles at T = 840, 1080, and 1320. Also, in this case we assume that $K_s = 0.0185$, $K_\theta = 1.5 \times 10^{-4}$, $\alpha = 1.8$, $c = 0.1$ and $\varphi_0 = 0°$.

Supplementary Note 7: Solitons excited by pulling

While in Supplementary Note 5, we consider the case in which condition (S36) is satisfied and the system supports two elastic vector solitons, here we investigate the response of the system when only one elastic vector soliton exists (i.e. when $D_1 > D_2$ or $D_1 < -D_2$). Let us consider the case $D_1 < -D_2$. Existence of such solitary wave requires that

$$C_1 > 0, \quad \frac{C_2^2}{9C_1^2} - \frac{C_3}{2C_1} > 0 \quad \text{and} \quad D_1 < -D_2. \tag{S49}$$

Substitution of Eqs. (S24) and (S28) into conditions (S49) yields,

$$\begin{aligned} &c^2 > \alpha^2 (K_s - K_\theta), \quad c^2 > \frac{3K_\theta}{2\sin^2\varphi_0 + 3K_\theta}, \\ &\text{and} \;\; c^2 > \frac{3K_\theta (7\cos(2\varphi_0) - 1)}{\cos(2\varphi_0)(21K_\theta + 8) - 2\cos(4\varphi_0) - 6 - 3K_\theta}. \end{aligned} \tag{S50}$$

Assuming that $\varphi_0 < 0.7137 = 41°$ (so that $7\cos(2\varphi_0) > 1$), condition $(\text{S50})_3$ is only satisfied if

$$\cos(2\varphi_0)(21K_\theta + 8) - 2\cos(4\varphi_0) - 6 - 3K_\theta < 0 \tag{S51}$$

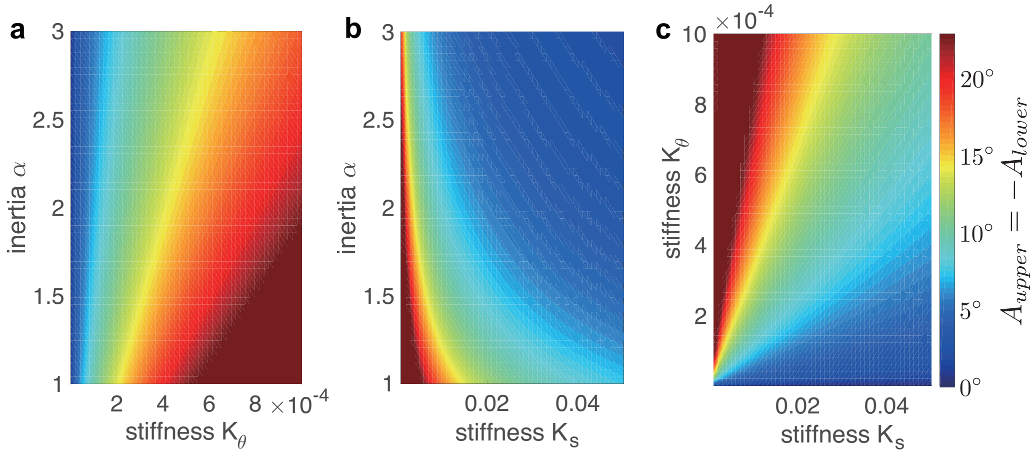

Supplementary Figure 14: (a) Evolution of $A_{upper} = -A_{lower}$ as a function of α andK_θ (assuming $K_s = 0.02$). (b) Evolution of $A_{upper} = -A_{lower}$ as a function of α andK_s (assuming $K_\theta = 1.5 \times 10^{-4}$). (c) Evolution of $A_{upper} = -A_{lower}$ as a function of K_θ andK_s (assuming $\alpha = 1.8$).

since positive values of the denominator always lead to $c > 1$. In Supplementary Figure 15a, we report the region of the φ_0-K_θ domain in which condition (S51) is satisfied as the shaded area. We find that this type of solitary waves is supported by the system only for large enough φ_0.

Conditions (S50)$_{1,2}$ clearly show that there is a lower limit for the velocity of the propagating solitary waves, i.e.

$$c > c_{min} \tag{S52}$$

where

$$c_{min} = \max\left\{ \alpha\sqrt{K_s - K_\theta},\ \sqrt{\frac{3K_\theta}{2\sin^2\varphi_0 + 3K_\theta}} \right\}. \tag{S53}$$

When constraint (S52) is satisfied, the system supports only one solution in the form

$$\theta = \frac{1}{D_1 - \ D_2 \cosh\left(\zeta/W\right)} \tag{S54}$$

where D_1 and D_2 are defined in Eqs. (S28) and (S31). This solution is plotted in Supplementary Figure 15b, assuming $K_s = 0.0185$, $K_\theta = 1.5\times10^{-4}$, $\alpha = 1.8$, $c = 0.5$ and $\varphi_0 = 8°$. Moreover, in Supplementary Figure 15c we report the displacement component, obtained via Eq. (S32). We notice that, in contrast to the cases considered in the previous sections, the displacement is negative and has a positive gradient, indicating that the soliton stretches the structure in the longitudinal direction during propagation (note that all solitons considered in previous sections induce compressive stresses within the chain). As such, these solitary waves can be excited by pulling one end of the chain.

To verify the validity of our analytical solution, in Supplementary Figure 15d-e we compare the analytical solutions to numerical results obtained by direct integration of the full discrete

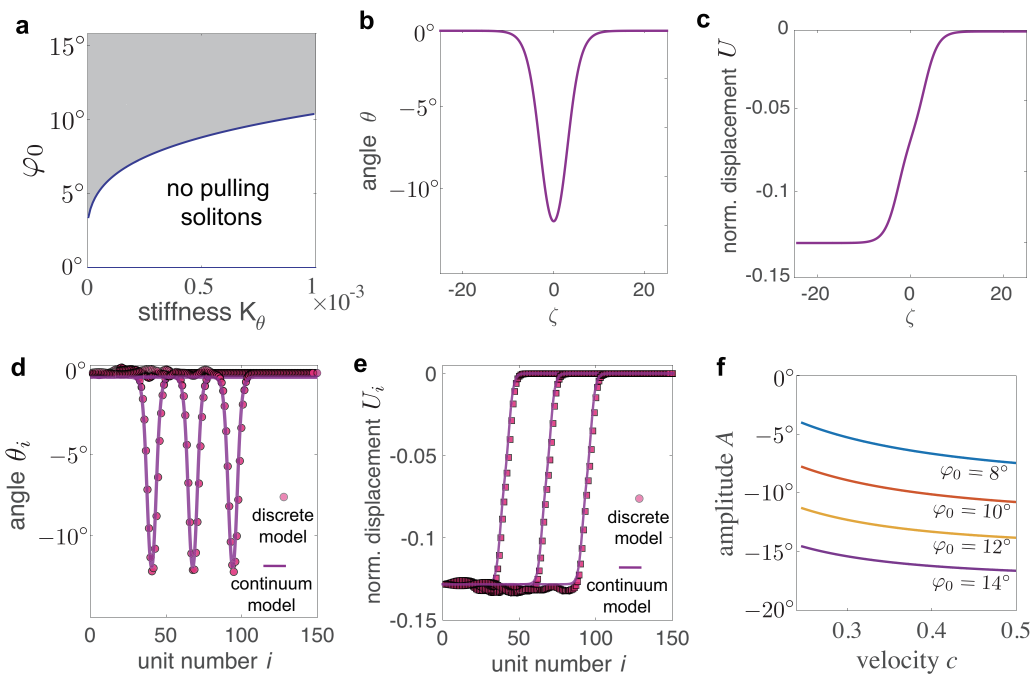

Supplementary Figure 15: (a) Region (shaded area) of the φ_0-K_θ domain in which pulling solitons exist. (b)-(c) Analytical solution for a structure characterized by $K_s = 0.0185$, $K_\theta = 1.5 \times 10^{-4}$, $\alpha = 1.8$, $c = 0.5$ and $\varphi_0 = 8°$. (d)-(e) Comparison between analytically (lines) and numerically (markers) predicted rotation and normalized displacement on the same structure at T = 226, 294, and 360. (f) Relation between pulse velocity and amplitude for pulling solitons propagating in structures characterized by different angles φ_0.

model (Eqs. S3). Note that in this set of simulations we consider a chain with 2×150 crosses and input our theoretical solutions (i.e. Eqs. (S54)) as boundary conditions for the first pair of crosses at the left end of the chain, while implementing free-boundary conditions at its right end. We find an excellent agreement between our analytical (lines) and numerical (markers) results, confirming the validity of our continuum model. Finally in Supplementary Figure 15f, we present the c-A relation for solitary waves excited by pulling. Unlike the case of compressive solitons, which propagate faster for smaller amplitudes, we find that the ones with larger amplitude travel faster (i.e. c always increases when the amplitude A increases). This difference is due to the fact that under compression our system exhibits nonlinear strain softening, while under tension it exhibits strain hardening. Finally, we note that we expect pulling solitons to be difficult experimentally observe, since they require an excitation applied at a very high speed.

Supplementary Note 8: Energy carried by solitons

In this section we determine the energy carried by the elastic vector solitons supported by our

system. To begin with, we focus on the i-th pair of crosses, whose total energy is given by the sum of the elastic energy stored in the hinges and the kinetic energy of two rigid crosses. As shown in Supplementary Figure 16, there are two types of hinges associated to the i-th pair of crosses: two horizontal hinges (whose deformation is characterized by $\delta^i_{l_H}$, $\delta^i_{s_H}$ and $\delta^i_{\theta_H}$) and one vertical hinge (whose deformation is characterized by $\delta^i_{l_V}$, $\delta^i_{s_V}$ and $\delta^i_{\theta_V}$).

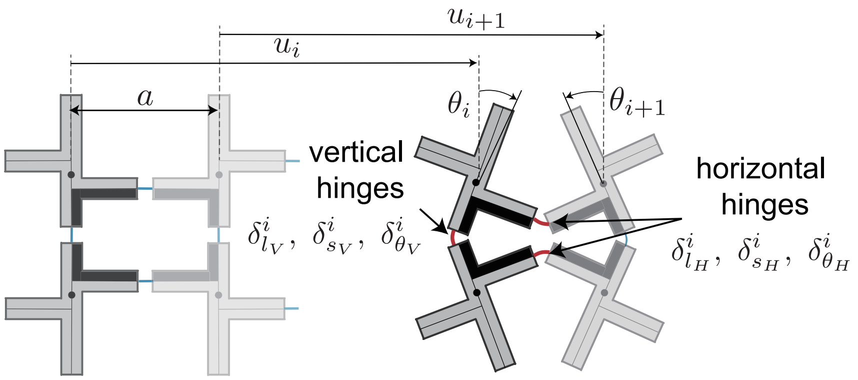

Supplementary Figure 16: A schematic diagram for the analysis of unit cell energy.

It follows that the total energy for the i-th pair of crosses can be expressed as,

$$e_i = \frac{1}{2}k_l(2\delta^{i\,2}_{l_H} + \delta^{i\,2}_{l_V}) + \frac{1}{2}k_s(2\delta^{i\,2}_{s_H} + \delta^{i\,2}_{s_V}) + \frac{1}{2}k_\theta(2\delta^{i\,2}_{\theta_H} + \delta^{i\,2}_{\theta_V}) + m\left(\frac{\partial u_i}{\partial t}\right)^2 + J\left(\frac{\partial \theta_i}{\partial t}\right)^2, \quad \text{(S55)}$$

where

$$\begin{aligned}
\delta^i_{l_H} &= u_{i+1} - u_i + \frac{a}{2\cos\varphi_0}\big[2\cos\varphi_0 - \cos(\varphi_0+\theta_i) - \cos(\varphi_0+\theta_{i+1})\big],\\
\delta^i_{s_H} &= \frac{a}{2\cos\varphi_0}\big[\sin(\varphi_0+\theta_{i+1}) - \sin(\varphi_0+\theta_i)\big],\\
\delta^i_{\theta_H} &= \theta_{i+1} + \theta_i,\\
\delta^i_{l_V} &= \delta^i_{s_V} = 0,\\
\delta^i_{\theta_V} &= 2\theta_i.
\end{aligned} \quad \text{(S56)}$$

Eq. (S55) can be written in dimensionless form as

$$E_i = \frac{e_i}{k_l a} = \Delta^{i\,2}_{l_H} + K_s\Delta^{i\,2}_{s_H} + \frac{K_\theta}{8\cos^2\varphi_0}(2\delta^{i\,2}_{\theta_H} + \delta^{i\,2}_{\theta_V}) + \left(\frac{\partial U_i}{\partial T}\right)^2 + \frac{1}{4\alpha^2\cos^2\varphi_0}\left(\frac{\partial \theta_i}{\partial T}\right)^2 \quad \text{(S57)}$$

where $\Delta^i_* = \delta^i_*/a$ are the normalized deflections

$$\begin{aligned}
\Delta^i_{l_H} &= U_{i+1} - U_i + \frac{1}{2\cos\varphi_0}\big[2\cos\varphi_0 - \cos(\varphi_0+\theta_i) - \cos(\varphi_0+\theta_{i+1})\big],\\
\Delta^i_{s_H} &= \frac{1}{2\cos\varphi_0}\big[\sin(\varphi_0+\theta_{i+1}) - \sin(\varphi_0+\theta_i)\big].
\end{aligned} \quad \text{(S58)}$$

Next, we introduce the continuum functions $\theta(X,T)$ and $U(X,T)$ and assume that the width of the propagating waves is much larger than the unit cell size and that $\theta \sim \varphi_0 << 1$. The total energy of the i-th pair of crosses (Eq. (S57)) can then be approximated as

$$\begin{aligned} E(X,T) = &\left(\frac{\partial U}{\partial X} + \frac{\cos\varphi_0 - \cos(\varphi_0+\theta)}{\cos\varphi_0}\right)^2 + \frac{K_s}{4}\left(\frac{\partial \theta}{\partial X}\right)^2 + \frac{3K_\theta \theta^2}{2\cos^2\varphi_0} \\ &+ \left(\frac{\partial U}{\partial T}\right)^2 + \frac{1}{4\alpha^2\cos^2\varphi_0}\left(\frac{\partial \theta}{\partial T}\right)^2, \end{aligned} \tag{S59}$$

which can be rewritten in terms of the traveling coordinate $\zeta = X - cT$ as

$$\begin{aligned} E(\zeta) = &\left(\frac{\partial U}{\partial \zeta} + \frac{\cos\varphi_0 - \cos(\varphi_0+\theta)}{\cos\varphi_0}\right)^2 + \frac{K_s}{4}\left(\frac{\partial \theta}{\partial \zeta}\right)^2 + \frac{3K_\theta \theta^2}{2\cos^2\varphi_0} \\ &+ c^2\left(\frac{\partial U}{\partial \zeta}\right)^2 + \frac{c^2}{4\alpha^2\cos^2\varphi_0}\left(\frac{\partial \theta}{\partial \zeta}\right)^2 \end{aligned} \tag{S60}$$

Finally, the energy carried by the elastic vector solitons is given by

$$E_S = \int_{-\infty}^{+\infty} E(\zeta) d\zeta, \tag{S61}$$

where U and θ entering in Eq. (S60) are defined by Eqs. (S29) and (S32).

In Supplementary Figure 17 we report the evolution of E_S as a function of the amplitude A as predicted by Eq. (S61) for the aligned (i.e. $\varphi_0 = 0$) and shifted (i.e. $\varphi_0 = 5°$) chains.

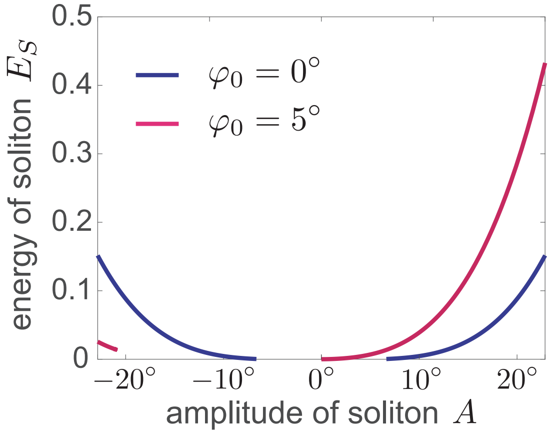

Supplementary Figure 17: Evolution of E_S as a function of the amplitude A for the aligned (blue line) and shifted (purple line) chains.

Supplementary Note 9: Dispersion relation

In this section we linearize the discrete governing equations of the system (Eqs. (S3)) to derive its dispersion relation. We start by assuming that θ_{i+p} (with $p = 0, \ \pm 1$) is small, so that

$$\begin{aligned} \sin(\theta_{i+p} + \varphi_0) &\approx \sin(\varphi_0) + \cos(\varphi_0)\theta_{i+p}, \\ \cos(\theta_{i+p} + \varphi_0) &\approx \cos(\varphi_0) - \sin(\varphi_0)\theta_{i+p}. \end{aligned} \tag{S62}$$

Substitution of Eqs. (S62) into Eqs. (S3) yields the linearized equations,

$$\begin{aligned} \frac{\partial^2 U_i}{\partial T^2} =& U_{i+1} - 2U_i + U_{i-1} + \frac{1}{2}\tan(\varphi_0)\left(\theta_{i+1} - \theta_{i-1}\right), \\ \frac{1}{\alpha^2}\frac{\partial^2 \theta_i}{\partial T^2} =& \left(K_s\cos^2(\varphi_0) - \sin^2(\varphi_0) - K_\theta\right)\left(\theta_{i+1} + \theta_{i-1}\right) \\ & - 2\left(K_s\cos^2(\varphi_0) + \sin^2(\varphi_0) + 2K_\theta\right)\theta_i - \sin(2\varphi_0)\left(U_{i+1} - U_{i-1}\right), \end{aligned} \tag{S63}$$

which can be written in matrix form as

$$\mathbf{M}\ddot{\mathbf{\Phi}}_i + \sum_{p=-1,0,1} \mathbf{K}^{(p)}\mathbf{\Phi}_{i+p} = 0, \tag{S64}$$

where

$$\begin{aligned} &\mathbf{M} = \begin{bmatrix} 1 & 0 \\ 0 & \alpha^{-2} \end{bmatrix}, \quad \mathbf{\Phi}_{i+p} = \begin{bmatrix} U_{i+p} \\ \theta_{i+p}, \end{bmatrix}, \\ &\mathbf{K}^{(0)} = \begin{bmatrix} 2 & 0 \\ 0 & 2\left(K_s\cos^2(\varphi_0) + \sin^2(\varphi_0) + 2K_\theta\right) \end{bmatrix}, \\ &\mathbf{K}^{(\pm 1)} = \begin{bmatrix} -1 & \mp\tan(\varphi_0) \\ \pm\sin(2\varphi_0) & -\left(K_s\cos^2(\varphi_0) - \sin^2(\varphi_0) - K_\theta\right) \end{bmatrix}. \end{aligned} \tag{S65}$$

Next, we seek a solution of Eqs. (S64) in the form of a harmonic wave

$$\mathbf{\Phi}_i(T) = \tilde{\mathbf{\Phi}}_i(\mu)\exp\imath(\mu X_i - \omega T), \tag{S66}$$

where ω is the normalized cyclic frequency of harmonic motion (the dimensional cyclic frequency is $\omega\sqrt{k_l/m}$), μ is the wavenumber, $\imath = \sqrt{-1}$ and $\tilde{\mathbf{\Phi}}_i = [\tilde{U}_i, \ \tilde{\theta}_i]$ is a vector that defines the amplitude of wave motion. Substitution of Eq. (S66) into Eq. (S64) yields

$$-\omega^2\mathbf{M}\tilde{\mathbf{\Phi}}_i + \sum_{p=-1,0,1} \mathbf{K}^{(p)}\tilde{\mathbf{\Phi}}_{i+p}e^{\imath\,\mu\,X_{i+p}} = 0, \tag{S67}$$

with $X_{i+p} = i + p$. Eq. (S67) is an eigenvalue problem that yields two dispersion branches, $\omega^{(1)}(\mu)$ and $\omega^{(2)}(\mu)$, each corresponding to a linear wave mode. While for the general case

$\varphi_0 \neq 0$ Eq. (S67) can only be solved numerically to find $\omega^{(1)}$ and $\omega^{(2)}$, for the special case $\varphi_0 = 0$ an analytical solution exists

$$\begin{aligned} \omega^{(1)} &= \sqrt{2 - 2\cos\mu}, \\ \omega^{(2)} &= \alpha\sqrt{2(K_s + 2K_\theta) - 2(K_s - K_\theta)\cos\mu}. \end{aligned} \tag{S68}$$

with the corresponding eigenvectors given by

$$\tilde{\mathbf{\Phi}}^{(1)} = \left[1,\ 0\right]^T \text{ and } \tilde{\mathbf{\Phi}}^{(2)} = \left[0,\ 1\right]^T \tag{S69}$$

In Supplementary Figure 18a and b we report the dispersion relation for a chain with $\varphi_0 = 0°$ and $5°$, respectively. Note that each point on the dispersion curves is color-coded to show the corresponding modal polarization, which for the k-th branch is defined as

$$\eta_\theta^{(k)} = \frac{\tilde{\theta}_i^{(k)}}{\sqrt{\left(\tilde{\theta}^{(k)}\right)^2 + \left(\tilde{U}_i^{(k)}\right)^2}}\ \text{, with } k = 1,\ 2. \tag{S70}$$

We find that the linear modes of the structure with $\varphi_0 = 5°$ contain both rotational and translational component (i.e., the two degrees of freedom are coupled). Differently, for the special case $\varphi_0 = 0°$ the two propagation modes are fully decoupled, each associated with a single degree of freedom (i.e. since $\eta_\theta = 0$ on the first branch and $\eta_\theta = 1$ on the second one, the corresponding modes are purely translational and rotational, respectively). We also find that for the case $\varphi_0 = 0°$ the purely rotational mode branch starts at a finite frequency (i.e. linear waves with rotational component are not supported for $\omega < \alpha\sqrt{6K_\theta}$), indicating that the system possesses a linear low-frequency band gap for rotation (highlighted in green in Supplementary Figure 18a). To demonstrate the effect of such linear frequency gap, we numerically solve the ODEs given by Eqs. (S3) for a chain with 150 pairs of crosses excited by a longitudinal displacement $U_{input} = 10^{-4}\sin\omega T$ applied to the mid-point at its left end. In Supplementary Figure 18c and d we report the numerically recorded transmittance for both rotational and translational component, $\max(\theta_{100}(T))/\max(\theta_2(T))$ and $\max(U_{100}(T))/\max(U_2(T))$. We find that for the case $\varphi_0 = 0$ the transmission of rotational modes significantly drops at low frequencies, confirming the existence of the low-frequency band gap for rotations. By contrast, no such drop in transmission is observed for the system with $\varphi_0 = 5°$, a consequence of the absence of any type of gap.

Lastly, it is important to note that the frequency content of the solitary waves supported by our system overlaps with this low-frequency band gap for rotation (see Supplementary Figure 18e for a typical soliton characterized by $A = 0.30$). As discussed in the main text and in Supplementary Note 9, the behavior of our soliton splitters is affected by such overlap, which prevents radiation of rotational vibrations.

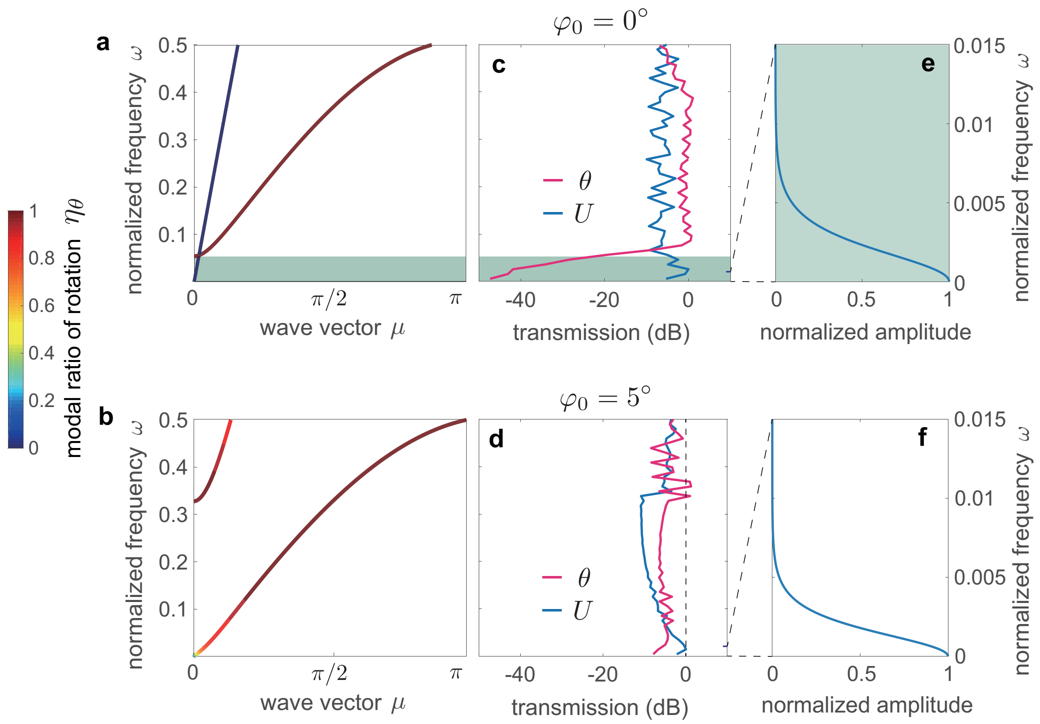

Supplementary Figure 18: (a)-(b) Linear dispersion relations for a chain with $\varphi_0 = 0$ and $\varphi_0 = 5°$, respectively. The dispersion curves are color-coded to show the modal ratio of rotation η_θ. (c)-(d) Amplitude transmission coefficients of the two linear modes for a chain with $\varphi_0 = 0$ and $\varphi_0 = 5°$, respectively; (e)-(f) Frequency content of a typical soliton supported by a chain with $\varphi_0 = 0$ and $\varphi_0 = 5°$, respectively. Note that the frequency range is changed in order to show the low frequency dominance in the soliton.

Supplementary Note 10: Additional results for splitter

In this Section, we report the results of additional numerical analysis and experiments conducted on our soliton splitter.

Experimental results

While in the main text we show experimental results for a soliton splitter with a pair of stiffer hinges made of polyester sheets with thickness $t_h^d = 0.635$ mm (so that $K_s^d/K_s = K_\theta^d/K_\theta = 30$), in Supplementary Figure 19 we present experimental results for our 2×50 sample with $\varphi_0 = 0$ and two stiffer hinges made of polyester sheets with thickness $t_h^d = 0.508$ mm (Supplementary Figure 19a) and $t_h^d = 0.635$ mm (Supplementary Figure 19b) and paper clips (Supplementary Figure 19c), which connect the 24th and the 25th pairs of crosses. Note that these three pairs of stiffer hinges result in $K_s^d/K_s = K_\theta^d/K_\theta = 18$, 30 and 175, respectively. The results clearly indicate that the amount of reflected and transmitted energy can be controlled by varying the stiffness ratio. For the splitter characterized by $K_s^d/K_s = K_\theta^d/K_\theta = 18$, most of the energy of the incident pulse is carried by the transmitted soliton and only a small portion by the reflected one; for the splitter characterized by $K_s^d/K_s = K_\theta^d/K_\theta = 30$, as described in main text, the energy carried by the incident pulse is roughly equally split between the transmitted and reflected solitons; for the splitter characterized by $K_s^d/K_s = K_\theta^d/K_\theta = 175$, most of the energy of the incident pulse is carried by the reflected soliton.

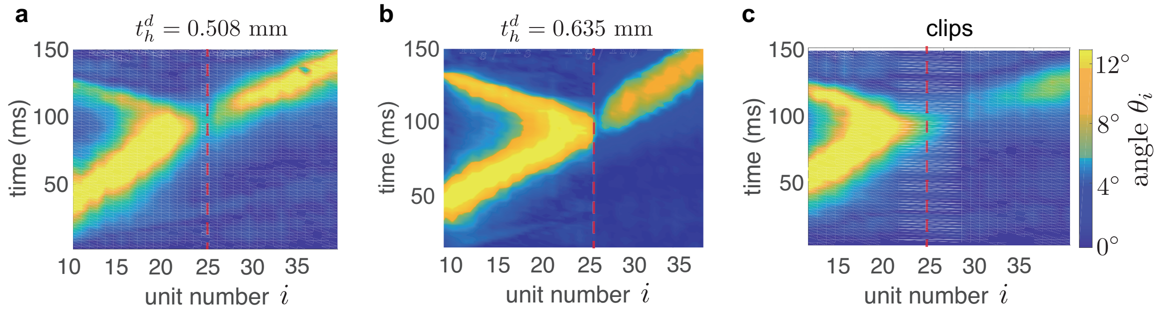

Supplementary Figure 19: Experimentally measured rotation of the pairs of crosses during the propagation of the pulse for soliton splitters with two stiffer hinges made of (a) polyester sheets with thickness $t_h^d = 0.508$ mm, (b) polyester sheets with thickness $t_h^d = 0.635$ mm and (c) paper clips. The location of the stiff pair of hinges is indicated by the dashed red line

Finally, in Supplementary Figure 20 we compare the experimentally measured rotation during the propagation of the pulse in a chain characterized by $\varphi_0 = 0°$ (Supplementary Figure 20a) and $\varphi_0 = 5°$ (Supplementary Figure 20b). Note that in both cases the sample comprises 2×50 units and a stiffer pair of hinges made of polyester sheets with thickness $t_h^d = 0.635$ mm is introduced to connect the 24th and the 25th pairs of crosses. While in the aligned chain, the pulse excited at the left end of the chain is split into two clean solitons by the pair of stiffer

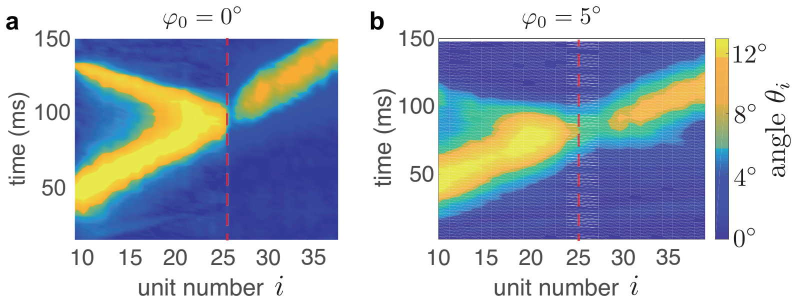

Supplementary Figure 20: Experimentally measured rotation during the propagation of the pulse for soliton splitters characterized by (a) $\varphi_0 = 0°$ and (b) $\varphi_0 = 5°$. The location of the stiff pair of hinges (made of polyester sheets with thickness $t_h^d = 0.635$ mm) is indicated by the dashed red line

hinges, in the shifted chain the signal both transmitted and reflected by the stiffer pair of hinges is much more noisy.

Numerical results

While in Fig. 3 of the main text we present results only for the rotational component θ, in Supplementary Figure 21 we show also the evolution of U as the solitary wave propagates in a 2×1000 chain characterized by $\varphi_0 = 0$ with a pair of stiffer hinges (with $K_s^d/K_s = K_\theta^d/K_\theta = 30$) connecting the 500th and 501st rigid crosses. The spatio-temporal plots for both θ (Supplementary Figure 21a) and U (Supplementary Figure 21c) clearly indicate that the pair of stiffer hinges splits the incoming soliton into two pulses that propagate with stable shape and constant velocity and that no trains of solitons are generated. As for the radiation of linear waves, we find that the interaction between the soliton and the stiffer hinges generate only translational vibrations (see small fluctuations in Fig Supplementary Figure 21c), since the overlap between the frequency content of the solitary waves supported by our system and the low-frequency band gap for rotation (see Supplementary Figure 18) prevents radiation of small amplitude rotational waves. We also note that the energy transferred to the translational vibrations can be estimated by comparing the energy carried by the incident soliton to that carried by the reflected and transmitted ones. Since in the aligned chain (i.e. for $\varphi_0 = 0$) the amplitudes of the incident, reflected and transmitted solitons are measured to be $A_i = 17.2°$, $A_r = 13.75°$ and $A_t = 14.89°$ (see Supplementary Figure 21a and b), using Eq. (S61) and the results of Supplementary Figure 17 we find that the normalized energy carried by them is $E_{S,i} = 0.0465$, $E_{S,r} = 0.0184$ and $E_{S,t} = 0.0262$, respectively. Hence, since in our splitter $(E_{S,r} + E_{S,t})/E_{S,i} = 0.96$, only 4% of the energy carried by the incoming soliton is transferred to translational linear vibrations. By contrast, for the shifted structure (for which $\varphi_0 = 5°$) we measure $A_i = 16.45°$, $A_r = 5.45°$ and $A_t = 12.08°$ (see Supplementary Figure 21e and f), so that $E_{S,i} = 0.1575$, $E_{S,r} = 0.008$ and $E_{S,t} = 0.064$. As such, in the shifted structure, which lacks both an amplitude gap for solitons and a low-frequency band gap for linear rotational

vibrations, $\sim 54\%$ of the energy carried by the incoming solitary wave is transferred to small amplitude solitons and linear vibrations. Finally, it is important to point out that the group velocity of the longitudinal vibrations supported by the chain characterized by $\varphi_0 = 0$ is one magnitude larger than the typical velocity of solitons (i.e. the solitons propagating in the chain have velocity $c \sim 0.1$, whereas the velocity of the longitudinal vibration in the long wavelength limit is found from the dispersion relation of Supplementary Figure 18a to be ~ 1). As such, the linear waves propagating in our splitter are very fast and do not interfere with the transmitted and reflected solitary waves, leading to a clean splitter for solitons.

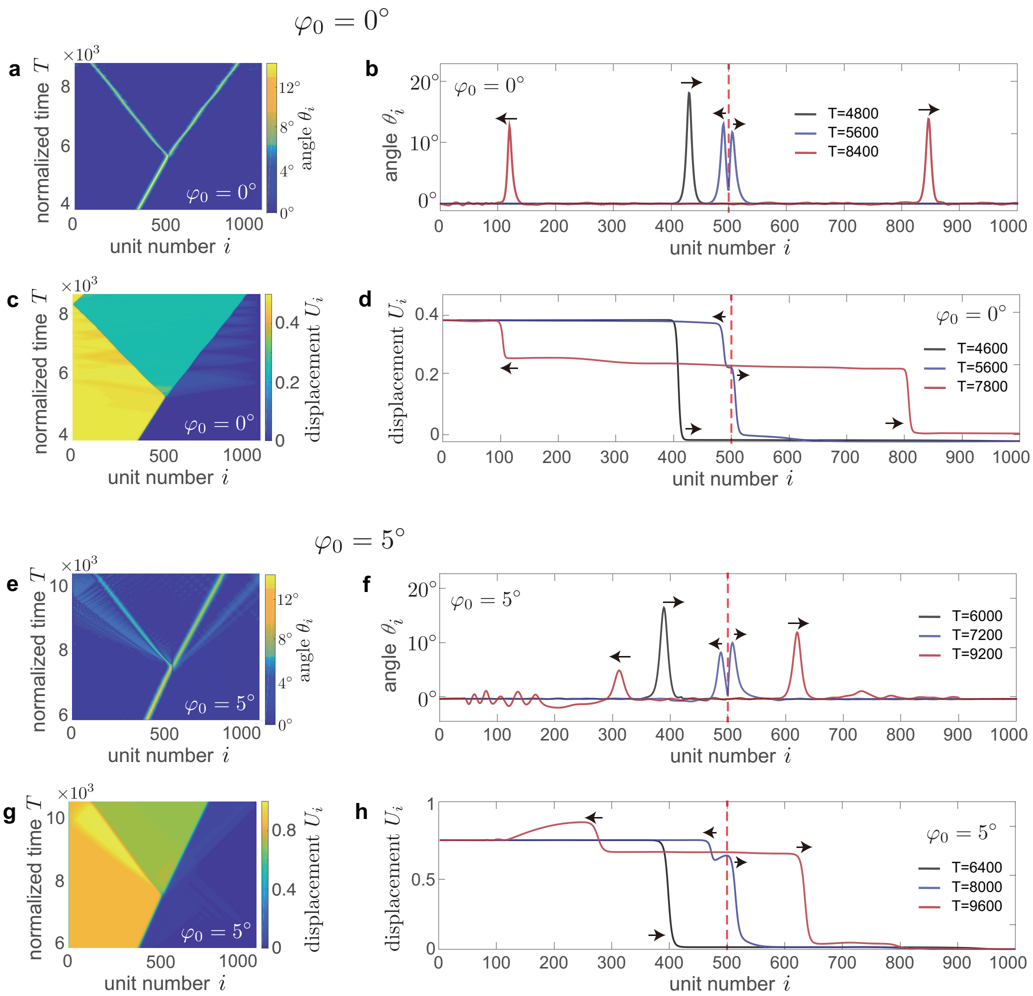

Supplementary Figure 21: Soliton splitter with a stiff defect ($K_s^d/K_s = K_\theta^d/K_\theta = 30$) between the 500th and 501st rigid crosses . (a)-(d) Numerical results for a 2×1000 chain with symmetric crosses characterized by $\varphi = 0°$. (e)-(h) Numerical results for a 2×1000 chain with asymmetric crosses characterized by $\varphi = 5°$.

While the results reported in Supplementary Figure 21 are for a chain with a stiffer hinge, we also conducted an additional numerical simulation on a chain that embeds a softer hinge.

More specifically, we considered a chain comprising 1000 units and a pair of softer hinges (with $K_s^d/K_s = K_\theta^d/K_\theta = 1/30$) connecting the 500th and 501st rigid crosses. The results shown in Supplementary Figure 22a-d indicate that in the system with $\varphi_0 = 0$ the fragmented oscillations are wiped out due to the amplitude gap and that only a small amount of purely translational vibrations are generated by the interaction between the soliton and the softer pair of hinges. Differently, for the shifted chain ($\varphi_0 = 5°$) due to the absence of both the amplitude gap for solitary waves (i.e. $A_{upper} = 0°$ for the system) and the low-frequency linear gap for rotational vibrations, trains of pulses and linear vibrations are generated when the propagating soliton hits the pair of softer hinges.

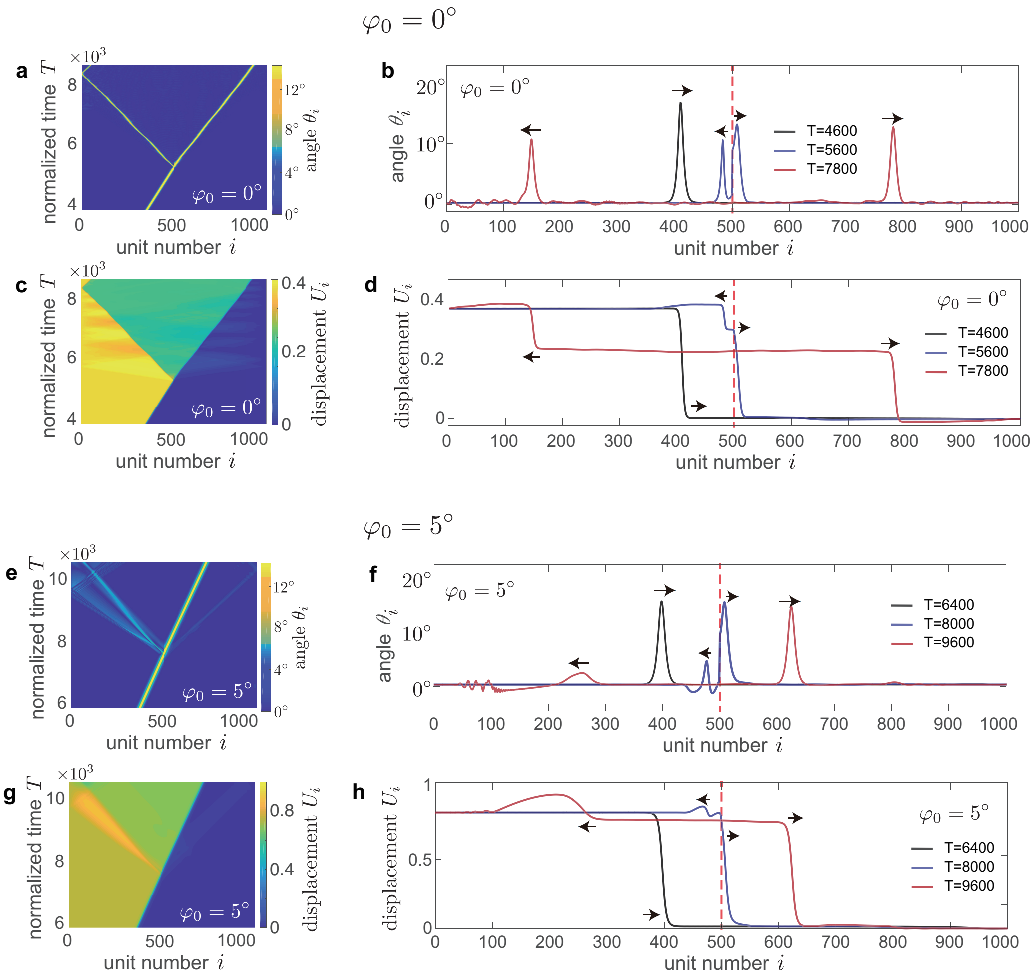

Supplementary Figure 22: Soliton splitter with a softer defect ($K_s^d/K_s = K_\theta^d/K_\theta = 1/30$) between the 500th and 501st rigid crosses . (a)-(d) Numerical results for a 2×1000 chain with symmetric crosses characterized by $\varphi = 0°$. (e)-(h) Numerical results for a 2×1000 chain with asymmetric crosses characterized by $\varphi = 5°$.

Finally, we conduct a set of discrete numerical simulations on an aligned chains to further explore the effect of the stiffness ratio $K_s^d/K_s = K_\theta^d/K_\theta$ on the response of the system. In

all our simulations we consider an aligned chains (i.e. $\varphi_0 = 0°$) comprising 2×1000 crosses and a pair of stiffer hinges (with stiffness K_s^d and K_θ^d) connecting the 500th and 501st units. In

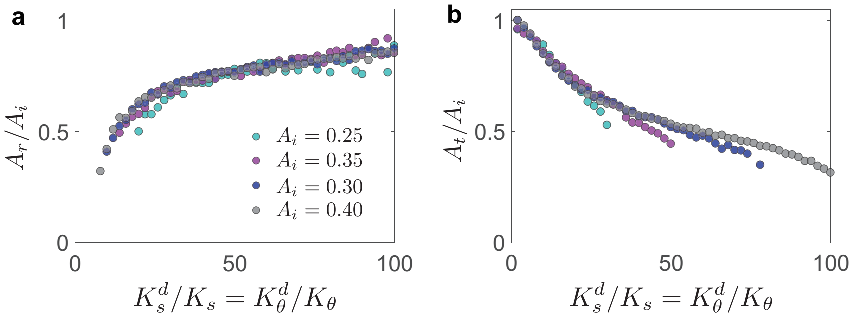

Supplementary Figure 23: Numerical results showing the effect of $K_s^d/K_s = K_\theta^d/K_\theta$ on the amplitude of the transmitted and reflected solitons. Amplitude ratios (a) A_r/A_i and (b) A_t/A_i as a function of $K_s^d/K_s = K_\theta^d/K_\theta$ for different values of A_i. The splitter considered in our simulations comprises a chain of 2×1000 crosses with $\varphi_0 = 0°$, $K_s = 0.02$, $K_\theta = 1.5 \times 10^{-4}$, $\alpha = 1.8$ and $\varphi = 0°$. A pair of stiffer hinges are inserted at the center of the chain with stiffness ratio $K_s^d/K_s = K_\theta^d/K_\theta \in [1,\ 100]$.

our simulations we consider $K_s^d/K_s = K_\theta^d/K_\theta \in [1,\ 100]$ and input our theoretical solutions (i.e. Eqs. (S29) and (S32)) as boundary conditions for the first pair of crosses at the left end of the chain, while implementing free-boundary conditions at its right end. We consider input signal of different magnitude (A_i), identify the normalized time T_c at which the pulse is split by the stiffer pair of hinges and then monitor the amplitude of the reflected (A_r) and transmitted (A_t) solitons at $T_c + 1000$. In Supplementary Figure 23a and b we show the amplitude ratios A_r/A_i and A_t/A_i for $A_i = 0.25, 0.30, 0.35$ and 0.40. We find that: (i) the amplitude of the reflected soliton A_r increases monotonically with $K_\theta^d/K_\theta = K_s^d/K_s$; ($ii$) the amplitude of the transmitted soliton A_t decreases monotonically for increasing $K_\theta^d/K_\theta = K_s^d/K_s$; ($iii$) Both A_r/A_i and A_t/A_i do not depend on the amplitude of the input signal, suggesting that our soliton splitter is a robust device.

Supplementary Note 11: Additional results for diode

In this Section, we provide additional experimental and numerical results for our mechanical diode.

Experimental results

In Fig. 4 of the main text we report the spatio-temporal rotation diagrams for two of the experiments we conducted on our mechanical diode. In Supplementary Figure 24 we show the experimental results for the remaining five experiments.

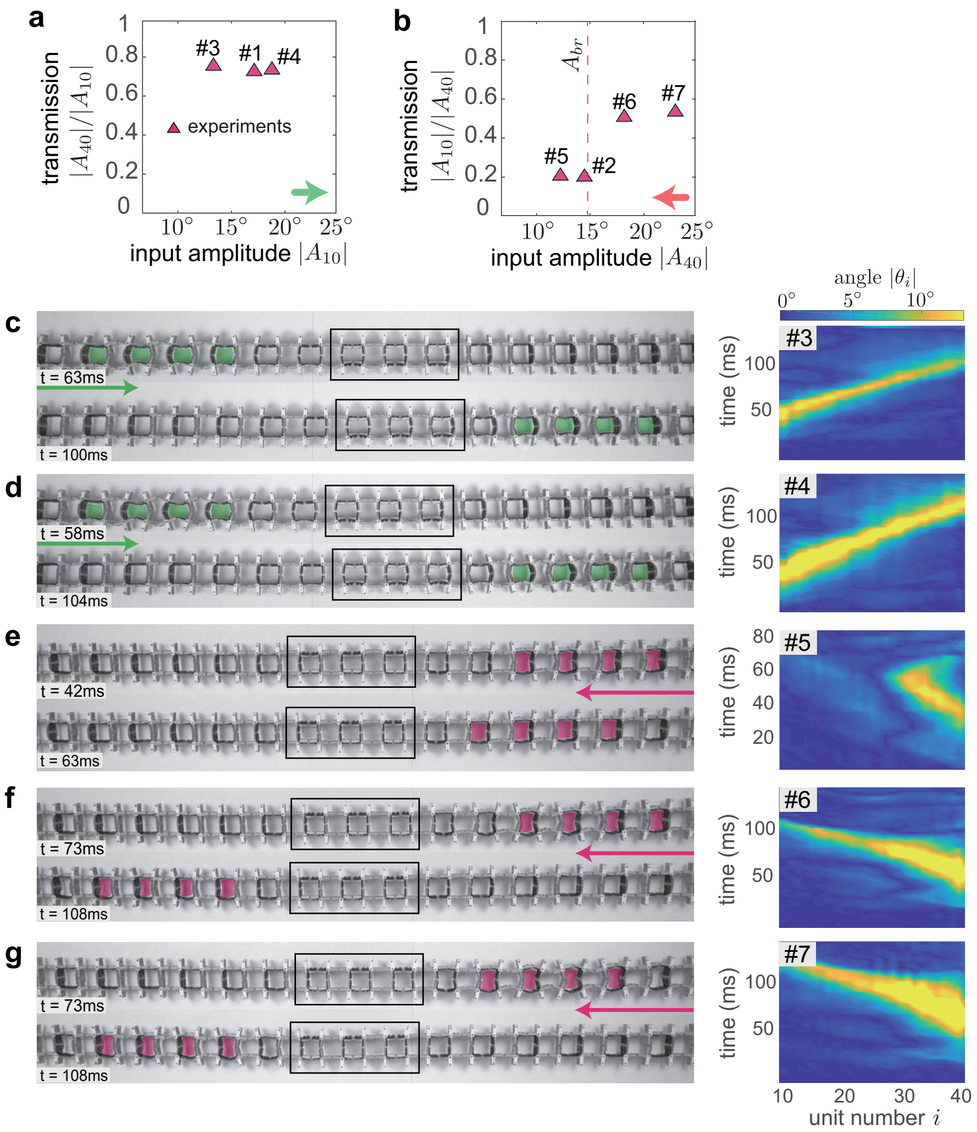

Supplementary Figure 24: (a) Experimentally measured transmission, $|A_{40}|/|A_{10}|$, as a function of the input amplitude, $|A_{10}|$, for pulses excited at the left end of the chain. (b) Experimentally measured transmission, $|A_{10}|/|A_{40}|$, as a function of the input amplitude, $|A_{40}|$, for pulses excited at the right end of the chain. (c)-(g) Optical images and corresponding rotation of the pairs of crosses as measured in five different experiments.

Numerical results

In the main text, we have investigated the response of a mechanical diode with $2N$ and $(2N+1)$ pairs of crosses characterized by $\varphi_0 = 0$ and a central portion consisting of $2N_a$ pairs of crosses with $\varphi_0 = 5°$, assuming $N = 12$ and $N_a = 3$ (see Fig. 4 of the main text). We found that for all pulses with amplitude larger than $A_{upper}^{\varphi_0=0} = 6.55°$ initiated at the left end of the system the

transmission, $|A_{40}|/|A_{10}|$ approaches unity. Differently, when the excitation is applied at the right end of the chain, the transmission $|A_{10}|/|A_{40}|$ is close to zero, even if the amplitude of the input signal is outside the gap of the region with $\varphi_0 = 0$ (i.e. $|A_{40}| > A_{upper}^{\varphi_0=0}$). However, as typically observed in electronic [6] and thermal [7] diodes, if the amplitude of the pulses becomes too large, the diode experiences a condition known as breakdown. As a result, solitary waves with amplitude larger than $A_{br} \approx 15°$ propagate through the diode (i.e. if $|A_{40}| > A_{br} \approx 15°$, then $|A_{10}|/|A_{40}| \sim 0.6$). It is important to note that the breakdown amplitude A_{br} is analogous to the breakdown voltage of electronic diodes and determines the reliability of the device, since it defines the largest amplitude of a soliton that the diode can block.

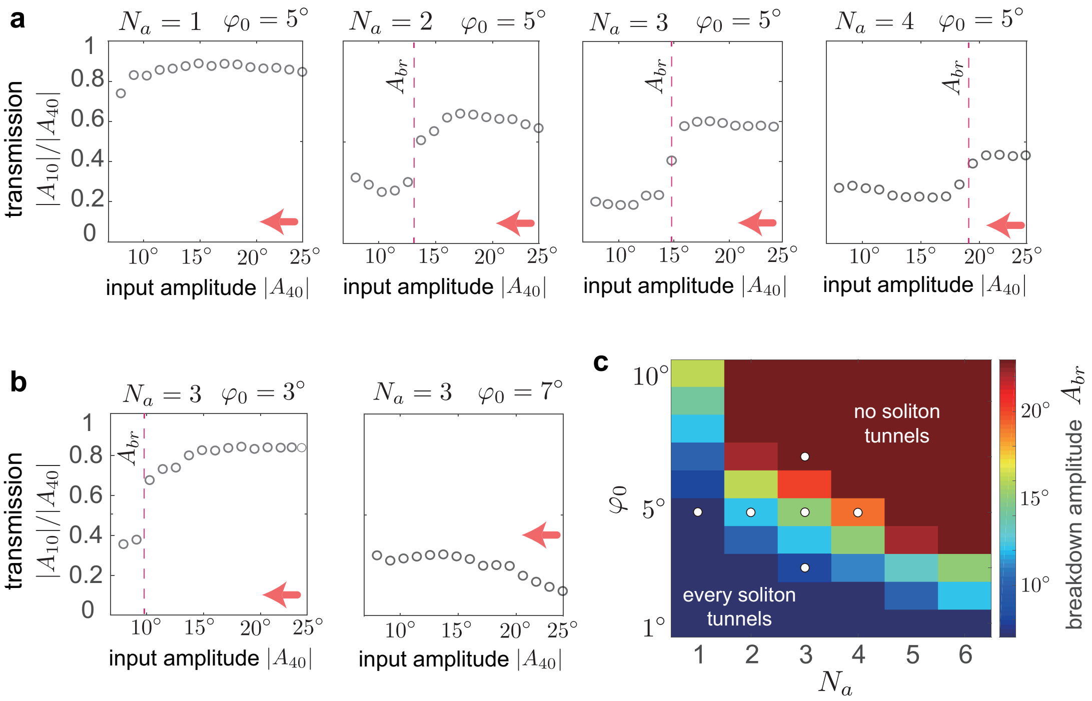

Supplementary Figure 25: Numerical results showing the effect of N_a and φ_0 on the breakdown amplitude A_{br}. (a) Transmission as a function of the amplitude of the input signal for diodes characterized by different numbers N_a of pairs of crosses with $\varphi_0 = 5°$ (while keeping N=12). (b) Transmission as a function of the amplitude of the input signal for diodes characterized by $N_a = 3$ and $\varphi_0 = 3°$ and $7°$ (while keeping N=12). (c) Evolution of the breakdown amplitude A_{br} as a function of N_a and φ_0.

To study the effect of φ_0 and N_a on A_{br}, we conduct a set of discrete simulations in which we excite pulses of different amplitude at the right end of the chain and measure the transmission $|A_{10}|/|A_{40}|$. In Supplementary Figure 25a we report the measured transmission as a function of the amplitude of the input signal for diodes characterized by different values of N_a and $\varphi_0 = 5°$ (while keeping N=12). We find that as N_a increases, A_{br} becomes larger, indicating a longer shifted section enhances the performance of our diode. We also note that for $N_a = 1$, the transmission is always close to unity and our structure no longer functions as a diode. In fact,

every incident soliton large enough to propagate through the aligned chain tunnels through such a diode.

In Supplementary Figure 25b we report the measured transmission as a function of the amplitude of the input signal for diodes characterized by $N_a = 3$, $\varphi_0 = 3°$ and $7°$, and N=12. We find that lower values of φ_0 make the diode less reliable, since A_{br} decreases ($A_{br} \approx 15°$ for $\varphi_0 = 5°$ and $A_{br} \approx 10°$ for $\varphi_0 = 3°$). Differently, for $\varphi_0 = 7°$, all pulses considered in the simulations are blocked by the diode and no solitons tunnel through.

Finally, in Supplementary Figure 25c we summarize all of our numerical results and report the evolution of A_{br} as a function of N_a and φ_0.

Supplementary References

[1] M. Senn, *https://www.mathworks.com/matlabcentral/ fileexchange/50994-digital-image-correlation-and-tracking* (2016).

[2] A. Polyanin, V. Zaitsev, *Handbook of Nonlinear Partial Differential Equations, Second Edition* (Chapman and Hall/CRC, 2011).

[3] B. Deng, J. R. Raney, V. Tournat, K. Bertoldi, *Phys Rev Lett* **118**, 204102 (2017).

[4] X. D. Cao, C. J. McKinstrie, *J Opt Soc Am B* **10**, 1202 (1993).

[5] Y. S. Kivshar, *J Opt Soc Am B* **7**, 2204 (1990).

[6] P. Horowitz, W. Hill, *The Art of Electronics*, Cambridge low price editions (Cambridge University Press, 1989).

[7] G. Wehmeyer, T. Yabuki, C. Monachon, J. Wu, C. Dames, *Appl Phys Rev* **4**, 041304 (2017).
