# Supplementary material for: Metamaterials with amplitude gaps for elastic solitons
Source: Nat Commun. 2018 Aug 24;9:3410. doi: 10.1038/s41467-018-05908-9 (PMC6109112; doi:10.1038/s41467-018-05908-9)
Supplement: Supplementary file 3 — Description of Additional Supplementary Files [file 41467_2018_5908_MOESM3_ESM.pdf]

Description of Additional Supplementary Files

File Name: Supplementary Movie 1
Description: Assembly of the aligned chain using LEGO bricks and plastic shims.

File Name: Supplementary Movie 2
Description: Excitation of elastic vector solitons in our system using a pendulum and an impactor.

File Name: Supplementary Movie 3
Description: Propagation of elastic vector solitons in the aligned chain.

File Name: Supplementary Movie 4
Description: Assembly of the shifted chain using LEGO bricks and plastic shims.

File Name: Supplementary Data 1
Description: Instruction to run the MATLAB code.

File Name: Supplementary Data 2
Description: Main MATLAB file to be run to simulate the dynamic response of an aligned chain.

File Name: Supplementary Data 3
Description: MATLAB function that provides the boundary conditions.

File Name: Supplementary Data 4
Description: MATLAB function that provides the ODEs to be solved.
