# Supplementary material for: Metamaterials with amplitude gaps for elastic solitons
Source: Nat Commun. 2018 Aug 24;9:3410. doi: 10.1038/s41467-018-05908-9 (PMC6109112; doi:10.1038/s41467-018-05908-9)
Supplement: Supplementary file 2 — Peer Review File [file 41467_2018_5908_MOESM2_ESM.pdf]

Editorial Note: This manuscript has been previously reviewed at another journal that is not operating a transparent peer review scheme. The manuscript was considered suitable for publication without further review at Nature Communications.
